# Retrieval of germinal zone neural stem cells from the cerebrospinal fluid of premature infants with intraventricular hemorrhage

**DOI:** 10.1002/sctm.19-0323

**Published:** 2020-05-30

**Authors:** Beatriz Fernández‐Muñoz, Cristina Rosell‐Valle, Daniela Ferrari, Julia Alba‐Amador, Miguel Ángel Montiel, Rafael Campos‐Cuerva, Luis Lopez‐Navas, María Muñoz‐Escalona, María Martín‐López, Daniela Celeste Profico, Manuel Francisco Blanco, Alessandra Giorgetti, Elena González‐Muñoz, Javier Márquez‐Rivas, Rosario Sanchez‐Pernaute

**Affiliations:** ^1^ Unidad de Producción y Reprogramación Celular (UPRC) Red Andaluza para el diseño y traslación de Terapias Avanzadas Sevilla Spain; ^2^ Grupo de Neurociencia aplicada Instituto de Biomedicina de Sevilla Sevilla Spain; ^3^ Department of Biotechnology and Biosciences University Milan‐Bicocca Milan Italy; ^4^ Centro de Transfusiones Tejidos y Células de Sevilla (CTTS) Sevilla Spain; ^5^ Departamento de Preclínica Red Andaluza de Diseño y Traslación de Terapias Avanzadas Sevilla Spain; ^6^ Fondazione IRCCS Casa Sollievo della Sofferenza Production Unit of Advanced Therapies (UPTA) San Giovanni Rotondo Italy; ^7^ Regenerative Medicine Program Bellvitge Biomedical Research Institute (IDIBELL); Program for Translation of Regenerative Medicine in Catalonia (P‐CMRC) Barcelona Spain; ^8^ Department of Cell Biology Genetics and Physiology, University of Málaga Málaga Spain; ^9^ Department of Regenerative Nanomedicine Andalusian Center for Nanomedicine and Biotechnology‐BIONAND Málaga Spain; ^10^ Networking Research Center on Bioengineering Biomaterials and Nanomedicine (CIBER‐BBN). Carlos III Health Institute (ISCIII) Spain; ^11^ Neurosurgery Department Hospital Virgen del Rocío Sevilla Spain; ^12^ Present address: Centre for Genomics and Oncological Research (GENYO) Granada Spain

**Keywords:** cerebrospinal fluid, germinal zone, intraventricular hemorrhage, neural stem cell, neurogenesis, premature infant

## Abstract

Intraventricular hemorrhage is a common cause of morbidity and mortality in premature infants. The rupture of the germinal zone into the ventricles entails loss of neural stem cells and disturbs the normal cytoarchitecture of the region, compromising late neurogliogenesis. Here we demonstrate that neural stem cells can be easily and robustly isolated from the hemorrhagic cerebrospinal fluid obtained during therapeutic neuroendoscopic lavage in preterm infants with severe intraventricular hemorrhage. Our analyses demonstrate that these neural stem cells, although similar to human fetal cell lines, display distinctive hallmarks related to their regional and developmental origin in the germinal zone of the ventral forebrain, the ganglionic eminences that give rise to interneurons and oligodendrocytes. These cells can be expanded, cryopreserved, and differentiated in vitro and in vivo in the brain of nude mice and show no sign of tumoral transformation 6 months after transplantation. This novel class of neural stem cells poses no ethical concerns, as the fluid is usually discarded, and could be useful for the development of an autologous therapy for preterm infants, aiming to restore late neurogliogenesis and attenuate neurocognitive deficits. Furthermore, these cells represent a valuable tool for the study of the final stages of human brain development and germinal zone biology.

## Graphical abstract

Germinal zone neural stem cells (Gz‐NSC) are isolated from the hemorrhagic cerebrospinal fluid of preterm infants with severe intraventricular hemorrhage. These cells express ventral and posterior forebrain markers, can be differentiated and do not cause tumors. Gz‐NSC represent a valuable tool for the development of new cell therapies and the study of human Gz biology.

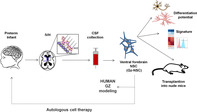

**Significance statement**

Intraventricular hemorrhage (IVH), occurring in 15% to 40% of preterm births, is frequently associated with long‐term neurological deficits. The rupture of the proliferative germinal zone in IVH disturbs late neuronal, ependymal, and glio‐genesis. Using a minimally invasive neuroendoscopic procedure, neural stem cells can be retrieved from the cerebrospinal fluid, which can be expanded, cryopreserved, and differentiated in vitro and in vivo, and are not tumorigenic. These cells display distinct hallmarks related to their origin in the germinal zone of the ventral forebrain and could be useful for the development of an autologous cell therapy aiming to attenuate neurocognitive sequelae.

## 1 INTRODUCTION

Intraventricular hemorrhage (IVH) is a common complication of premature infants, occurring in 15% to 40% of preterm infants weighing less than 1500 g at birth and being particularly common in extremely low birthweight neonates.^1^, ^2^, ^3^ IVH is classified into four grades according to the extent of hemorrhage, development of subsequent ventricular dilatation, and parenchymal involvement: grade I—a hemorrhage restricted to subependymal region; grade II—a hemorrhage bleeding into de ventricles without dilation; grade III—an IVH with ventricular dilatation; and grade IV—an IVH with associated adjacent brain parenchyma infarction.^4^ Some authors classify grade IV IVH separately because the presence of periventricular hemorrhagic infarction or other parenchymal lesions is generally not caused simply by extension of IVH into brain parenchyma and should thus be considered as a different pathological condition.^5^ Around 10% to 15% of preterm infants develop severe (grade III‐IV) IVH and those infants are at high risk to develop posthemorrhagic hydrocephalus, an expansion of the ventricles due to cerebrospinal fluid (CSF) accumulation (reviewed in Reference 6), and to present long‐term neurological deficits with cognitive and motor disabilities.^6^, ^7^ IVH initiates in the periventricular germinal zone (Gz), also known as germinal matrix, a highly proliferative, highly vascularized region around the lateral ventricles with a dense and fragile, endothelial‐lined, vessel network.^8^ From 24 to 32 weeks of gestation the Gz is most prominent in the caudo‐thalamic groove, forming the ganglionic eminences of the ventricular zone (VZ), where late migrating cortical and thalamic neurons and oligodendrocyte precursors are born.^9^ The ultimate cause of Gz bleeding remains unclear, but it is commonly accepted that it results from the combination of Gz vasculature vulnerability and blood pressure fluctuations associated with prematurity.^8^ In the hemorrhage phase, there is a rupture of the Gz, occurring most often at the level of the medial ganglionic eminence, that entails loss of neural stem cells (NSCs) and disturbs the cytoarchitecture of the zone leading to abnormal neuronal, ependymal, and glio‐genesis.^10^, ^11^ Current treatments for IVH are intended to decrease the intracranial pressure that can cause periventricular white matter compression and damage, impairment of brain development, and even death.^12^, ^13^ There is a standardized protocol neither for the type nor for the timing of the intervention,^14^, ^15^, ^16^ but it has recently been shown that early removal of hemorrhagic CSF by neuroendoscopic lavage is a safe procedure that may mitigate the adverse effects of the accumulation of blood products, decrease the need for permanent shunt placement, and potentially reduce neurological disability.^17^, ^18^, ^19^

NSCs are the self‐renewing, multipotent cells that generate neuronal and glial cell populations during development. During brain development, primary NSCs located in the VZ have radial processes (apical radial glial cells), divide symmetrically, are highly polarized, and express prominin‐1 (CD133).^20^ Radial glia are more multipotent than the intermediate progenitors of the subventricular zone (SVZ) that include basal radial glia, transient amplifying, and neural progenitors.^21^, ^22^ Regional differences in the transcriptional profile of radial glia dictate the fate of their postmitotic progeny. In addition to their differentiation potential, NSCs produce neurotrophic and neuroprotective molecules, making them attractive for regenerative approaches.^23^, ^24^ In this regard, clinical‐grade human NSC lines are usually obtained from the fetal central nervous system (CNS). Human NSCs can also be isolated from the adult CNS in patients undergoing surgical procedures,^25^ or be derived from pluripotent stem cells (PSC) and from somatic cells through reprogramming protocols.^24^, ^26^ Advanced therapy medicinal products based on clinical‐grade human allogenic fetal NSCs are being tested in clinical trials for various neurological disorders and, although efficacy has yet to be ascertained in a clinical setting, their safety profile has been repeatedly confirmed (reviewed in Reference 24).

Here we demonstrate that a novel class of NSCs can be robustly isolated from the hemorrhagic CSF of preterm neonates during neuroendoscopic lavage. These NSCs that we named Gz‐NCS display distinctive features corresponding to their origin in the ventral forebrain. Gz‐NSCs could be useful to develop an autologous cell therapy aiming to reduce neurological disability in preterm infants and to further our understanding of human Gz biology.

## 2 MATERIALS AND METHODS

### 2.1 Hemorrhagic CSF collection

CSF samples were obtained from eight preterm infants with grade IV IVH (Table 1) at the Hospital Universitario Virgen del Rocío (Sevilla, Spain). The study was approved by the Hospital Universitario Virgen del Rocío ethical committee and has been performed in accordance with the 1964 Declaration of Helsinki. All samples were obtained after parental informed consent. The SSPA Biobank has coordinated the collection, processing, management, and assignment of the biological samples used in this study, according to the standard operating procedures established for this purpose. Under intraoperative ultrasound guidance^27^ (Video S1) the ventricle with the larger amount of blood was punctured with the surgical endoscope (MINOPModular Neuroendoscopy system, Aesculap, Inc., Center Valley, Pennsylvania) and the content was collected. Briefly, the neurosurgical endoscopy system has two irrigation channels, a scope channel, and a working channel, allowing the simultaneous irrigation and collection of liquids during the surgical procedure. The endoscope is introduced through a minimal precoronal corticotomy, one channel is connected to an infusion line, and another channel to an outflow line open at the third ventricle level so that there is no positive resistance to liquid exit, being the system pressure close to 0 H_2_O cm; the infusion rate is determined by gravity (1.8 m) so that there is no risk of inadvertently causing hypertension during the procedure. Additionally, the neurosurgeon constantly monitors the fontanel tension and can open or close the lines to stabilize the pressures. The CSF is collected in 50 mL syringes connected to the outflow channel to minimize exerting an excessive suction. Once the initial hypertension is corrected, continuous irrigation is established using warm, lactated Ringer solution (Baxter, # FE2303) by passive inflow and outflow through the second channel (1.4 mm wide), which was also collected (lavage) in successive syringes until clear. Irrigation was stopped once the fluid within the ventricular system was clear, or at any time if hemodynamic instability appeared. Typically, 1000 to 2000 mL of Ringer solution were used and collected in 50 mL sterile syringes that were immediately closed to maintain sterility. The bleeding area was sealed with fibrinogen beads (Floseal, Baxter #ADS201845).

**TABLE 1:** Hemorrhagic cerebrospinal fluid samples

Batch	Weight (g)	Sex	EGA (weeks)
Case 1	2150	Male	33
Case 2	1950	Male	36
Case 3	1600	Male	30
Case 4	1142	Female	27
Case 5^a^	3120	Female	42
Case 6	1120	Male	29
Case 7	850	Male	29
Case 8	805	Female	28

a CD133 expression dropped drastically with passages and therefore cells isolated from this sample were excluded from further analysis.

Abbreviation: EGA, estimated gestational age at CSF collection.

### 2.2 NSC isolation from hemorrhagic CSF

CSF samples were transferred to appropriate tubes and centrifuged at 370*g* for 10 minutes. The cell pellet was resuspended in N2/B27 medium: Dulbecco's modified Eagle medium (DMEM)‐F12 (ThermoFisher Scientific #11530566), 0.1 mM nonessential amino acids (Sigma‐Aldrich #RNBG4911), 100 IU penicillin/100 μg/mL streptomycin (Sigma‐Aldrich #P0781), 2 μg/mL heparin (Rovi #641647), 1% N2 (ThermoFisher Scientific #11520536), 1X B27 (ThermoFisher Scientific #11530536), 20 ng/mL FGF (Miltenyi Biotec #130‐093‐564), 20 ng/mL EGF (Peprotech #AF‐100‐15), and 10 ng/mL LIF (Miltenyi Biotec #130‐108‐156). The cell suspension was seeded onto 20 μg/mL poly‐L‐ornithine (Sigma‐Aldrich # P4957)/20 μg/mL laminin from human placenta (Sigma‐Aldrich #L6274) (POL) or Matrigel (Corning #354277)‐coated plates. Medium was changed 24‐48 hours after seeding. Cells were seeded for expansion at 1 × 10^5^ cells/mL in low binding flasks or at 12 000 cells/cm^2^ in Matrigel‐ or POL‐coated plates. Matrigel‐coated flasks were prepared by incubation with Matrigel diluted in cold DMEM‐F12 for 1 hour at room temperature according to manufacturer's instructions. For POL coating, flasks were incubated with 20 μg/mL poly‐L‐ornithine for 1 hour at 37°C or overnight at 4°C. Flasks were washed twice with distilled water and they were then further incubated with 20 μg/mL laminin for 2 hours at 37°C. Flasks were washed three times with phosphate buffered saline (PBS, ThermoFisher Scientific #A12856‐01) before cell seeding. Cells were expanded for 3 (early) and 7‐10 (late) passages for characterization. Passage 7, which corresponds to 13 ± 1 accumulated population doublings, was considered “late passage” given that it will not be possible to extensively expand the cells in a clinical setting.

Magnetic activated separation (MACS) was performed using the CD133 MicroBead kit (Miltenyi Biotec #130‐097‐049) following manufacturer's instructions.

### 2.3 Immunofluorescence

Cells grown over Matrigel‐coated coverslips were fixed with 4% paraformaldehyde (SantaCruz Biotechnology #SC‐281692), permeabilized with 0.1% Triton X‐100 (Sigma‐Aldrich #T8787), blocked in PBS (ThermoFisher Scientific #A12856‐01) with 1% bovine serum albumin (BSA, Sigma‐Aldrich #A8806) for 30 minutes at 37°C and incubated with the primary antibody overnight at 4°C. Cells were subsequently incubated with the secondary antibody for 30 minutes at 37°C and mounted with ProLong Gold Antifade Mountant with 4′,6‐diamidino‐2‐phenylindole (DAPI, ThermoFisher Scientific #P36930). Primary and secondary antibodies are listed in Table S1. For Ki‐67 detection, we first performed an antigen retrieval step in which cells were heated for 10 seconds in a microwave with citrate buffer pH 6.0 (Sigma‐Aldrich #C9999) letting cells cool down 20 minutes. Acquisition of fluorescence images was performed in a Leica TCS‐SP5 or a Nikon Eclipse Ti fluorescence microscope. Images were processed using the Adobe Photoshop CS5 or ImageJ software.^28^ Positive cells were counted using the ImageJ software from at least three random fields per preparation.

### 2.4 Flow cytometry

For CD133, CD24, CD34, CD45, PODXL, IL1RAP, and MHC detection, live cells were blocked in PBS with 1% BSA and incubated with conjugated antibodies for 15 minutes at 4°C. For TREK2, FZD5, and DLK1 analysis, cells were fixed with 3.7% formaldehyde (Sigma‐Aldrich #F8775), permeabilized with 0.1% Triton X‐100 (Sigma‐Aldrich #T8787), blocked in PBS with 1% BSA and incubated with the primary antibody for 30 minutes at 4°C. Cells were subsequently incubated with the secondary antibody for 30 minutes at 4°C. Antibodies are listed in Table S1. Fluorescence was estimated with a Macs Quant flow cytometer (Miltenyi Biotec) and results were analyzed with the MacsQuantify 2.10 and FloJ v10 software. Appropriate isotype controls were run in parallel with the samples. Gating strategies for the different antibody panels are shown in Figure S1.

### 2.5 Transcriptomic analysis

#### 2.5.1 *Control stem cell samples*

Human fetal NSCs (n = 4) were derived from the forebrain of 15‐ to 22‐week‐old fetuses that had undergone spontaneous in utero death (miscarriage). Tissue procurement was approved by the Ethics Committee of the Institute “Casa Sollievo della Sofferenza” after receiving the mother's informed, written consent. These fetal NSC lines have been extensively characterized.^29^, ^30^, ^31^

PSC‐derived NSCs (iPS‐NSC, n = 6) were differentiated from embryoid bodies (EBs) in TeSR2 medium (Stemcell Technologies #05860) spiked with Rock inhibitor (Y‐7632; 10 μM; Tocris Bioscience #1254). After 7 days, EBs were plated on Matrigel and cultured in neural differentiation media. On day 10, 0.1 μM retinoic acid (RA, Sigma‐Aldrich #R 2625) was added to the medium. On day 15, neural tube‐like rosettes were mechanically detached and cultured in neural differentiation media with 20 ng/mL FGF (Miltenyi Biotec #130‐093‐564) and 20 ng/mL EGF (Peprotech #AF‐100‐15). Cells were expanded in suspension as neurospheres or in adhesion over Matrigel during 6 to 7 passages before RNA extraction for transcriptomic analysis.

Three human PSC lines were included for reference. CBiPS were derived from CD133^+^ umbilical cord cells. These cells are available from the Spanish national repository, (BNLC) and the data regarding cell characterization can be downloaded at http://www.eng.isciii.es. WA09 (H9) were purchased from WiCell and the characterization data can be downloaded at www.wicell.org. L6‐iPS was generated by our group (see experimental procedures in Supplementary Information) and we are in the process of banking it at the Spanish BNLC. Characterization of this cell line is provided in Figure S2.

Umbilical cord blood samples (CD34^+^ HSC, n = 3) were obtained from the Banc de Sang i Teixits, Barcelona. CD34^+^ cell purification was performed as previously described.^32^ Briefly, mononuclear cells (MNC) were isolated from CB using Lympholyte‐H (Cederlane #CL5015) density gradient centrifugation. CD34^+^ cells were positively selected using Mini‐Macs immunomagnetic separation system (Miltenyi Biotec #130‐046‐702,). Purification efficiency was determined by flow cytometry analysis staining with CD34‐phycoerythrin (PE; Miltenyi Biotec #130‐120‐520) antibody.

#### 2.5.2 *Expression microarrays*

RNA was extracted with RNeasy Mini kit (Qiagen #74104) following the instructions of the manufacturer and sent to the Genomics Unit of the Andalusian Center of Molecular Biology and Regenerative Medicine (CABIMER). RNA quality was analyzed by the Bioanalyzer 2100 (Agilent). All samples had RNA Integrity Number (RIN) higher than 9. cDNA was synthetized, labeled with biotin and hybridized with independent Human Clariom‐S Microarrays, (Affymetrix #902927) following Affymetrix protocol. Microarrays were scanned with Affymetrix GeneChip Scanner 3000 7G, and the obtained data were analyzed with the Affymetrix GeneChip Command Console 2.0 software. The microarray expression data set is publicly available at the GEO repository under the identifier GSE124361. Further analyses were performed using the Transcriptome Analysis Console (TAC, Affymetrix) v4.0 software and R version 3.5.0.^33^ Functional enrichment analysis was performed using the bioinformatics tool EnrichR (http://amp.pharm.mssm.edu/Enrichr/).^34^, ^35^ Neuroanatomical references were obtained from the Allen Atlas of the developing human brain (www.brainspan.org).^36^

#### 2.5.3 *RT‐PCR*

First, 0.1 μg of RNA were used for cDNA synthesis using Oligo‐dT (Invitrogen #18418012), RNase OUT (Invitrogen #10777‐014) and SuperScriptII Retrotranscriptase (Invitrogen #18064‐014). PCR products were obtained using 5 ng of cDNA and Mytaq Red DNA Polymerase (Bioline #BIO‐21108) following the manufacturer's instructions. Oligonucleotides used for amplification are provided in Table S2.

### 2.6 Transplantation into nude mice

Animal care and experimental procedures were conducted according to the current National and International Animal Ethics Guidelines and approved by the Italian Ministry of Health. Ten 7‐ to 8‐week‐old female athymic Nude‐Foxn1nu mice (#6902F, Envigo) received a single injection of 300 000 CD133+ cells in the striatum. Stereotaxic coordinates were determined using the Paxinos and Franklin atlas^37^ and the injections were done at: anteroposterior, 0 mm; dorsolateral, 2.5 mm from bregma; and dorsoventral, −2.7 mm from dura matter. All the injections were performed on the right hemisphere. Cells were injected in 3 μL during 5 minutes using HBSS (Gibco #14175095) as vehicle. Three animals were sacrificed at 3 weeks to assess survival and proliferation and seven animals were sacrificed at 6 months. Transplantation experiments and analyses were performed as previously described for fetal NSCs.^29^, ^38^ For immunohistochemical analysis, brains were perfused‐fixed with ice‐cold 4% paraformaldehyde (Sigma #158127), postfixed overnight, cryoprotected and sectioned in a cryostat. Twenty micrometers coronal sections were serially collected and processed for immunofluorescence.

Quantification of transplanted cells was done using Image J cell counter‐plugin on images acquired in a Nikon C2 confocal microscope and the NIS Elements 1.49 software. All cells expressing human nuclear antigen (huN) were counted in sections spanning the graft area, and the total number was calculated using Abercrombie correction^39^ and expressed as the percentage over injected cells. Migration was estimated as the distance between the first and last sections containing huN positive cells. Coexpression of Ki67, glial fibrillary acidic protein (GFAP), and OLIG2 was counted in three serial sections of the transplanted animals (n = 3‐7) and expressed as percentage over total huN^+^ cells in those sections.

### 2.7 Statistics

Data are presented as mean ± SEM. Significance was determined using two‐tailed Student's *t* test for comparisons between two samples. *P* < .05 was considered significant. Paired *t*‐tests or repeated measures (R) ANOVA were used to compare samples from the same individual at different stages. All statistical analyses were performed using the GraphPad Prism 8.01 software. Bioinformatic analyses were performed using the Affymetrix and R software, using *t*, ANOVA, and R‐ANOVA tests and selected thresholds, as indicated in the text and figure legends. A false discovery rate (FDR) <5% was established for significance.

## 3 RESULTS

### 3.1 Hemorrhagic CSF of preterm IVH patients contains NSCs

**FIGURE 1:**
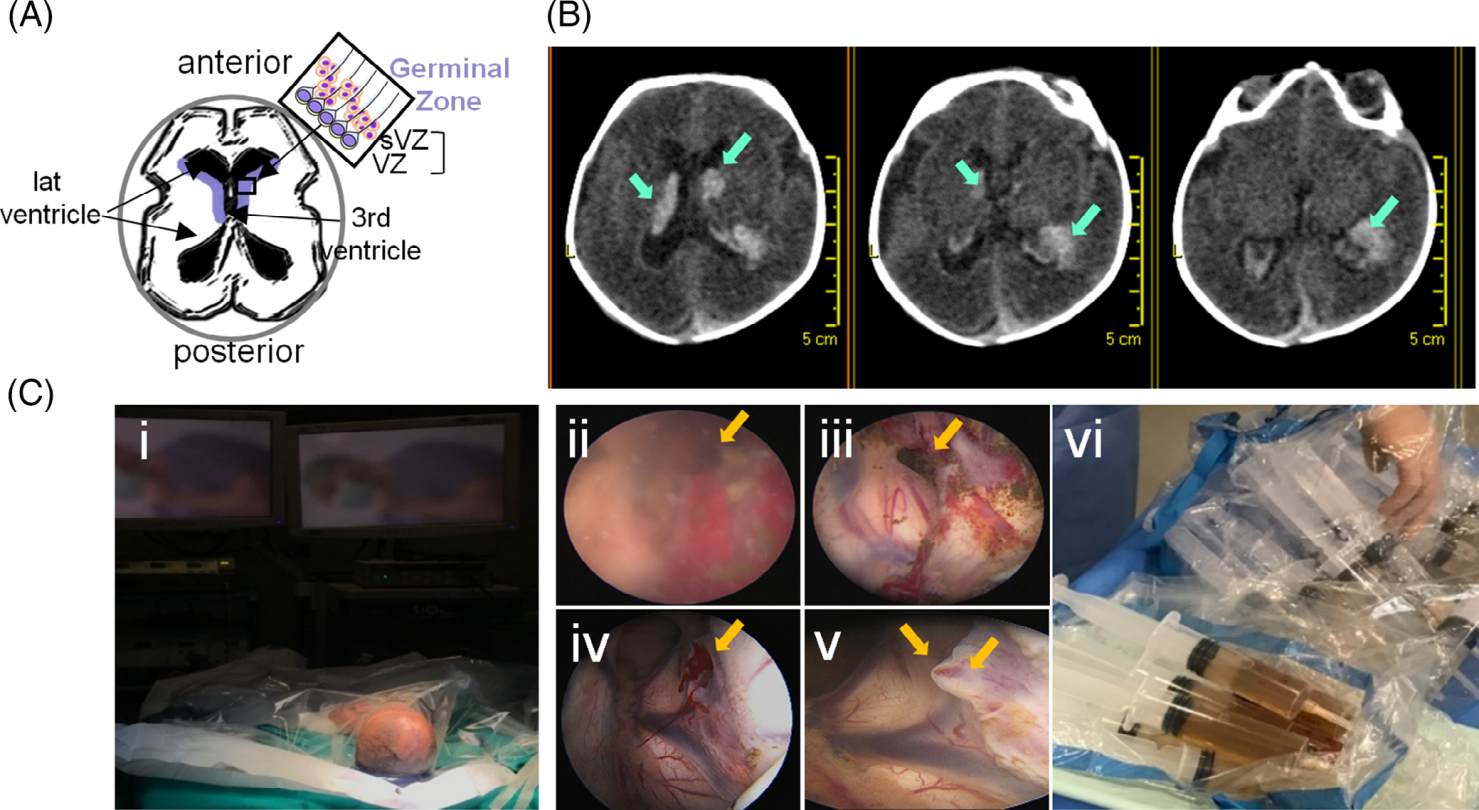
Neuroendoscopy and CSF collection. A, Schematic representation of the germinal zone localization (in blue) around the ventricles (axial view) that comprises the ventricular and subventricular zones where neural stem cells are found (box). B, Computed tomography axial brain images depicting the bleeding area close to the head of the caudate nucleus and the presence of blood inside the ventricular system (arrows) in one of the cases. C, Recovery of hemorrhagic CSF and irrigation fluid from preterm infants with IVH grade IV. Images of the surgical intervention by neuroendoscopy: preparation (i); neuroendoscopic imaging of bleeding area before (ii) and after (iii) irrigation, and, before (iv) and after (v) sealing; collection of irrigation fluid (vi)

**FIGURE 2:**
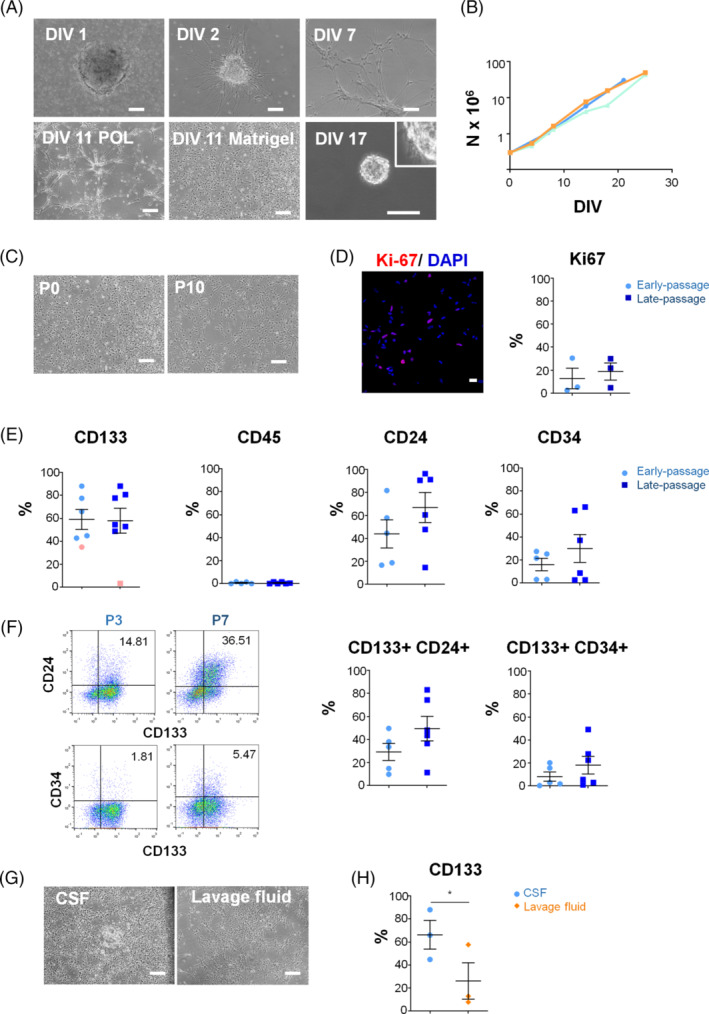
Isolation of NSC‐like cells from the CSF of IVH patients. A, Phase‐contrast microphotographs of CSF‐derived NSC cultures at different days in vitro (DIV) after isolation. Scale bar: 100 μm. B, Exponential growth kinetics of 3 representative NSC lines grown on Matrigel. C, Phase‐contrast microphotographs of cells at early (0) and late (10) passages grown on matrigel. D, Proliferation was assessed by quantification of Ki‐67 expression at early (3) and late (7) passages. A representative confocal section is shown. Scale bar 25 μm. E, Flow cytometry analysis of CD133, CD24, CD34 and CD45 at early (3) and late (7) passages. There were no significant differences between conditions. Data are shown as mean ± SEM of 5‐7 independent biological samples. The 42‐week‐old case (pink symbols) was excluded from further analysis. F, Co‐expression of CD133 with CD24 and CD34 at early and late passages. G, Representative microphotographs of the NSC‐like cells obtained from the CSF and the lavage fluid at 13 days after isolation. Scale bar: 100 μm. H, CD133 analysis by flow cytometry of the NSC‐like cells obtained from the CSF and the lavage fluid at passage 3. *P < .05

Eight consecutive cases with a clinical and radiological diagnosis of grade IV IVH (Table 1) underwent a ventricular neuroendoscopy to seal the bleeding area and remove the hemorrhagic CSF from the ventricular cavities (Figure 1, Video S1). Neuroendoscopic lavage was performed following the technique reported by Schulz et al^18^ with a few modifications as detailed in the Methods section. The content of the first syringe (collected before starting the irrigation) was processed for cell recovery. After centrifugation, the cell pellet was initially seeded on POL or Matrigel‐coated plates and cultured in an N2/B27 serum‐free medium with mitogens; 24‐48 hours after seeding, small aggregates were observed amidst abundant erythrocytes and blood cells in suspension (Figure 2A). Cells were enzymatically dissociated and passaged as neurospheres or in adhesion for 10 passages showing a doubling time of 3.94 ± 0.33 days and maintaining a stable growth curve and morphology (Figure 2B,C). Quantification of Ki‐67 showed no significant decrease in proliferation between early and late passages (12.77 ± 8.88 vs 18.83 ± 7.44; Figure 2D). Likewise, expression of the stem cell marker prominin‐1 (CD133) was maintained through passages (58.99 ± 8.7 vs 57.93 ± 10.82; Figure 2E). An exception was the 42‐weeks‐old sample (Figure 2E, pink symbols) in which the percentage of CD133^+^ cells dropped drastically upon passaging. This case was excluded from further analyses given that IVH in full‐term neonates most often originates in the choroid plexus.^8^

We next analyzed whether the cell population obtained from hemorrhagic CSF had a similar expression pattern of CD surface antigens than that described for fetal NSCs.^40^, ^41^ Like fetal NSCs, most cells in CSF samples were positive for CD133 and all were negative for CD45, displaying a variable expression of CD24 (Figure 2E and Table S3). Intriguingly, some samples contained a substantial percentage of CD34 positive cells (Figure 2E and Table S3), which is not expressed by fetal forebrain NSCs.^40^ On average, 18.10 ± 7.65% of the cells were CD133^+^CD34^+^ at passage 7, and 49.42 ± 10.65 of the cells were CD133^+^CD24^+^ at passage 7. Despite no significant differences, there was a trend to an increase of CD24 double positive cells with passages (29.13 ± 7.4 vs 49.42 ± 10.65; Figure 2F and Table S3).

We attempted to recover cells from subsequent CSF samples, collected once the irrigation started, but the flow cytometry analyses showed fewer CD133^+^ cells in the lavage fluid (66.26 ± 12.47 vs 26.18 ± 15.83; *P* = .02 [paired *t* test]; Figure 2G,H) so the rest of the study was carried out using only the first tube of hemorrhagic CSF from each case.

In another experiment, we failed to recover NSC‐like cells from nonhemorrhagic CSF samples (see supplementary experimental procedures and Figure S3) obtained from a different set of patients with obstructive hydrocephalus, although in some cases, changing to a serum‐based media allowed us to grow fibroblast‐like, adherent cells from samples with large CSF volumes (>20 mL).

### 3.2 NSCs isolated from the hemorrhagic cerebrospinal fluid present distinctive regional hallmarks

**FIGURE 3:**
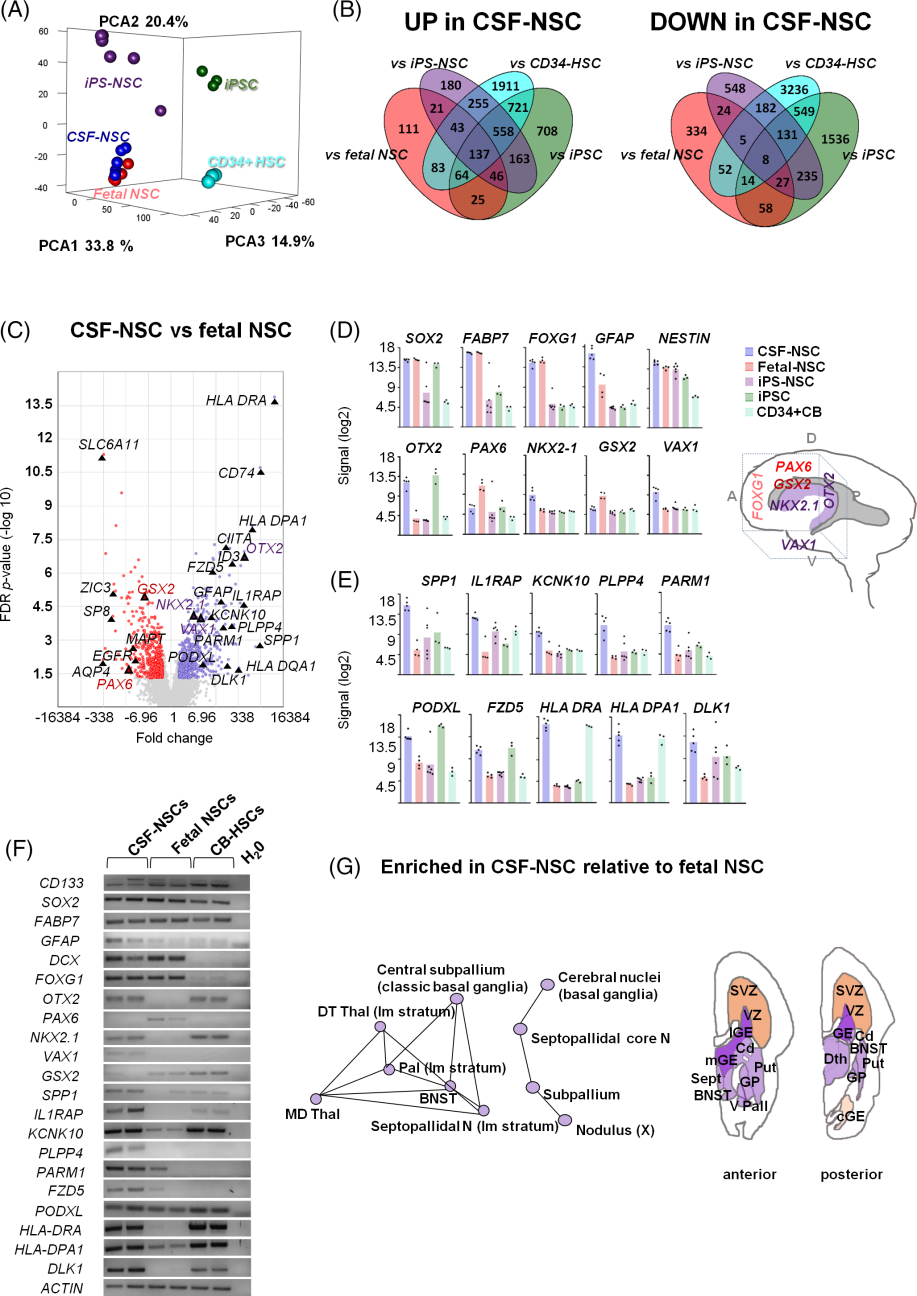
NSC cells isolated from the CSF display a ventral forebrain gene‐expression profile. A, PCA analysis of global gene‐expression profiles. B, Venn diagrams showing the number of differentially expressed genes (DEG), 2‐fold change, FDR *P* < .05. C, Volcano plot of DEG in NSCs from fetal brain (red) and CSF (blue) sources. Highlighted are markers that identify regional populations including genes that have been previously associated with germinal zones and forebrain regionalization (see also schematic in D) and putative candidates for prospective identification of germinal zone NSCs. Expression levels of NSC and regional forebrain markers (D) and candidate DEG genes that could identify this NSC population (E). F, Semi‐quantitative RT‐PCR of NSC markers and candidate DEG genes. G, Enrichment network analysis of upregulated genes relative to fetal NSCs, profiled across brain regions according to the Allen brain atlas, and schematic neuroanatomical representation on coronal brain sections showing their periventricular location. A, anterior; BNST, bed nuclei of the stria terminalis; cGE, caudal ganglionic eminence; Cd, caudate nucleus; D, dorsal; DThal, dorsal thalamus; GP, globus pallidus; lGE, lateral ganglionic eminence; mGE, medial ganglionic eminence; P, posterior; Put, putamen; Sept, septum; SVZ, subventricular zone; Thal, thalamus; V, ventral; V Pall, *ventral* pallidum; VZ, ventricular zone

We next performed a transcriptomic analysis to study the differences and similarities between NSCs isolated from hemorrhagic CSF, fetal forebrain NSCs and NSCs derived from iPSCs. Given that CSF samples contained mostly blood cells we also included in the analysis hematopoietic stem cells (CD34^+^ CB‐HSC) and the iPSC used to obtain iPS‐NSCs. Principal component analysis (PCA) mapping and hierarchical clustering of global gene‐expression profiles showed that NSCs from CSF clustered together with fetal NSCs, being farther away from iPS‐derived NSCs (Figure 3A). Pairwise comparisons showed a significant overlap in the expression profiles of the three types of NSCs (Figure 3B). Notwithstanding, there were 1073 differentially expressed genes (DEG) between CSF‐derived NSCs and fetal NSCs, using a false discovery rate (FDR) *P* value < .05 and ±2‐fold change (Figure 3C). Consistent with an NSC identity, expression of radial glia and neural progenitor markers, such as *SOX2*, *FABP7*, *FOXG1*, and *NES* was similar in CSF‐derived and fetal NSCs (Figure 3D). GFAP was highly expressed in both types, but significantly higher in the NSCs from CSF. GFAP expression is restricted to the VZ during primate brain development.^36^, ^42^ Likewise, other transcripts enriched in human VZ relative to the SVZ, (the secondary proliferative area) such as *SPP1*, *DLK1*, *IL1RAP*,^43^ or *ID3*, a marker of quiescent NSCs, were also higher in CSF than in fetal NSCs. On the other hand, expression of *EGFR*—which marks NSC activation—or regulators of lineage commitment, such as *AQP4*, *SP8*, or *ZIC3*, as well as more mature neuronal markers like *SOX1* or *MAPT* was higher in fetal forebrain NSCs (Figure 3C). There were also remarkable differences in the expression of forebrain regional transcription factors (Figure 3D) with high expression of ventral and posterior forebrain markers, *OTX2* and *NKX2.1*, while dorsal ones such as *PAX6* and *GSX2* were lower than in fetal NSCs. In addition, we identified several markers that could provide a distinctive signature for this NSC population (Figure 3C,E). Among those, there was a remarkable upregulation of genes related to antigen presentation and immune response, in particular pertaining to the major histocompatibility complex II (MHCII) (Figure 3E), which according to the developmental human brain atlas are highly expressed in germinal zones during mid‐gestational stages (www.brainspan.org).^36^ Differential expression of selected transcripts was validated by PCR (Figure 3F) Interestingly, enrichment analysis showed that the genes upregulated in CSF‐derived NSCs relative to fetal NSCs mapped to the ventral forebrain structures, including the periventricular nuclei—basal ganglia, thalamic, and septal nuclei (Figure 3G). This regional topography corresponds to the anatomical structures surrounding the ganglionic eminences, most often affected by IVH in preterm infants. This is consistent with an origin of the cells isolated from the CSF in the germinal zone of the ventral forebrain and therefore we have named them Gz‐NSCs.

**FIGURE 4:**
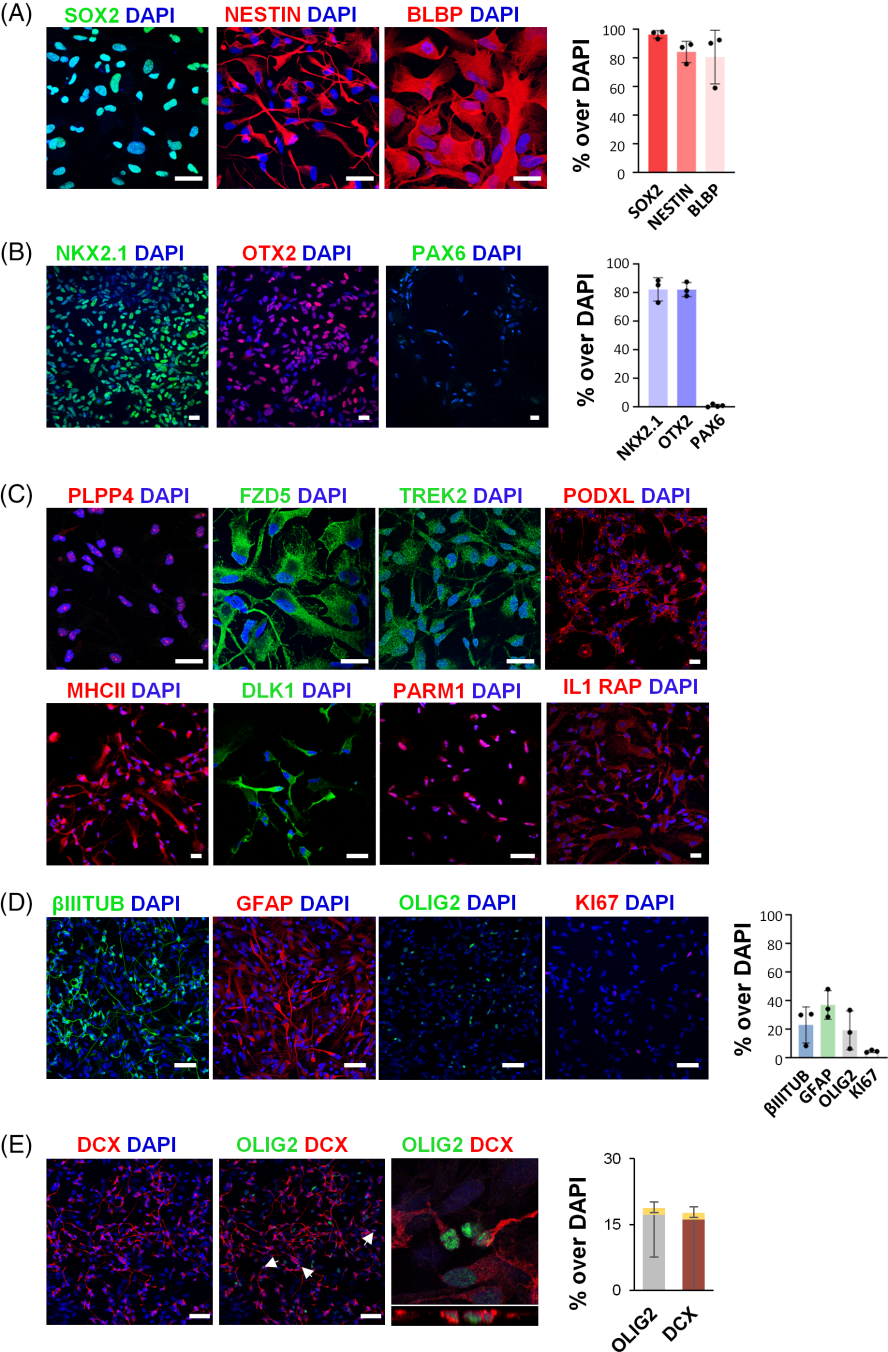
The cells isolated from hemorrhagic CSF display NSC features. Expression of NSC markers SOX2, Nestin, and BLBP (*FABP7*) (A) and regional markers NKX2.1, OTX2 and PAX6 (B) by immunofluorescence and corresponding quantification of at least 3 independent biological replicates. Scale bar: 25 μm. C, Immunofluorescence analysis of the expression of PLPP4, FZD5, TREK2, PODXL, MHC II, DLK1, PARM1, and IL1RAP in Gz‐NSCs. Representative confocal images of at least 3 independent biological replicates. Scale bar: 50 μm. D, Neural tri‐lineage differentiation into β‐III‐tubulin (βIIItub), glial fibrillary acidic protein (GFAP) and OLIG2 positive cells. Downregulation of Ki67 was detected upon differentiation. Representative confocal images (maximum projection) of 3 independent biological samples and corresponding quantification, shown as percentage over total cells. Scale bar: 50 μm. E, Confocal images and orthogonal z‐stack reconstruction showing colocalization of OLIG2 and doublecortin (DCX) (arrows) in a small percentage of the cells, represented in yellow in the bars. Scale bar: 50 μm

Next, we used immunofluorescence to evaluate the expression at the protein level of typical radial glia markers such as SOX2, nestin, and brain lipid binding protein (BLBP, *FABP7*). Quantification confirmed expression of these proteins by most cells (SOX2: 96.57 ± 1.56; NESTIN: 84.40 ± 4.27; BLBP: 80.80 ± 10.83; Figure 4A). In addition, the majority of the cells expressed regional transcription factors corresponding to ventral and posterior forebrain, NKX2.1 and OTX2, and not the dorsal forebrain marker PAX6 (NKX2.1:82.46 ± 4.73; OTX2: 82.33 ± 2.88; PAX6: 0.97 ± 0.48; Figure 4B). Other differentially expressed transcripts that we selected based on a putative membrane localization were also expressed at the protein level (Figure 4C). However, one of these, PLPP4, a poorly characterized phospholipid phosphatase expressed in the brain (www.proteinatlas.org)^44^ showed a clear nucleolar localization pattern.

To establish the differentiation potential of Gz‐NSCs, we cultured the cells in FBS without mitogens for 2 weeks. Cells showed in vitro tri‐lineage potential, upregulating neuronal, astrocyte, and oligodendrocyte markers in various proportions as shown in the graph (Figure 4D), and downregulating Ki67 (*P* < .01 compared with undifferentiated cells). Because OLIG2 is also expressed in neuronal progenitors, we examined the coexpression of OLIG2 and doublecortin (DCX). About 10% (9.9 ± 7) of the OLIG2 positive cells coexpressed DCX in these in vitro conditions, representing 1.5% of the total cells (Figure 4E). To further confirm differentiation into glial lineages, we also examined the expression of PDGFR, another oligodendrocyte marker, and S100β that was consistently coexpressed with GFAP in Gz‐NSCs differentiated in 2% B27 for 2 weeks (Figure S4).

### 3.3 CD133 ^+^ purified cells maintain Gz‐NSC features

**FIGURE 5:**
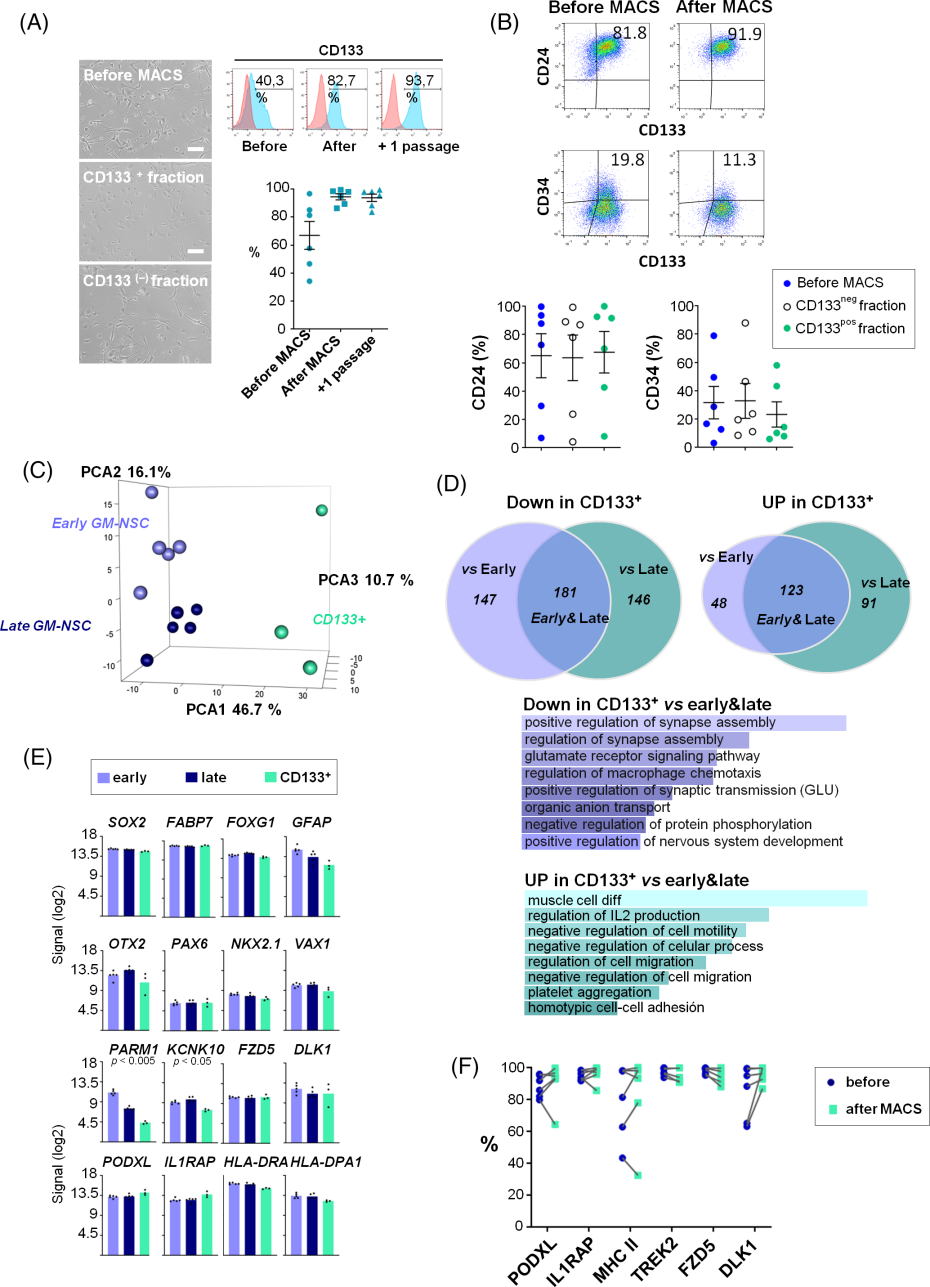
Gz‐NSC signature is maintained after CD133 sorting. A, Cell morphology and flow cytometry of CD133 after MACS purification. Results are the mean of 6 independent biological replicates. Scale bar: 100 μm. B, Flow cytometry analysis of CD24 and CD34 before and after purification for CD133. Percentages of CD24^+^ and CD34^+^ before sorting (blue) and in both the CD133 negative (white) and CD133 positive (green) fractions showed no significant differences. Data are shown as mean ± SEM of 6 independent biological samples. C, PCA analysis of early, late, and sorted Gz‐NSC populations. D, Venn diagrams representing the transcriptional changes related to cell propagation (early vs late) and CD133 sorting (2‐fold change, R‐ANOVA FDR F < 0.05). There were no DEG between early and late passages. Genes downregulated in the CD133 sorted cells with respect to early and late passages corresponded to GO pathways related to neuronal and synaptic activity while those upregulated in the sorted cells were indicative of a less differentiated stage. E, Expression of NSCs, regional and Gz‐NSC markers at the RNA level in early, late, and CD133^+^ purified cells. F, Flow cytometry analysis of PODXL, IL1RAP, MHC II, TREK2, FZD5, and DLK1 expression before and after MACS purification. Data are shown as mean ± SEM of 6 independent biological samples

Cells initially isolated from hemorrhagic CSF samples are a heterogeneous mixture of cellular types at different developmental and maturation stages, in particular taking into account that all these cases had parenchymal involvement (grade IV). Therefore, in order to better define putative Gz‐NSC‐specific features and obtain a more homogenous population for future in vivo applications, we selected CD133^+^ cells by magnetic activated cell sorting (MACS). CD133 has been used for the isolation of NSCs from normal brain tissues and CD133^+^ cells differentiate in vitro and in vivo into the three neuroectodermal lineages.^40^, ^45^, ^46^, ^47^ Following MACS purification, we could expand and cryopreserve the cells, which maintained their typical morphology and the expression of CD133 (Figure 5A). The percentages of CD34^+^ and CD24^+^ double positive cells were variable and did not significantly change with sorting, although we observed that CD34^+^ cells tended to remain in the negative fraction (Figure 5B).

We examined transcriptomic changes related to in vitro propagation and CD133 purification comparing early, late, and CD133^+^‐sorted Gz‐NSCs (R‐ANOVA, FDR F < 0.05) with no significant DEG between early and late passages (Figure 5C). Nevertheless, although there were no DEG at FDR < 0.05 between early and late passages, they appeared to be segregated along PCA2 (*y*‐axis, Figure 5C), therefore we further analyzed the data using a less stringent cutoff (Figure S5). Those analyses showed that, upon passaging, markers corresponding to more mature phenotypes tended to decrease, without weakening the NSC ventral identity (Figure S5).

Upon purification, there were significant changes relative to both early and late passage cells. Enrichment analysis of the common genes (early and late vs CD133^+^) showed that the expression of genes implicated in neural and synaptic specific pathways was decreased (Figure 5D). On the other hand, CD133^+^‐sorted cells showed a relative enrichment in genes expressed at less differentiated stages and in less specific pathways (Figure 5D). Transcriptomic analysis confirmed that CD133^+^ cells maintained the expression of radial glia and NSC markers as well as the pattern of regional transcription factors. Most of the putative membrane markers that we had selected, including genes related to antigen presentation, were also expressed (Figure 5E). In contrast, *PARM1* expression was significantly decreased and no longer different from fetal NSCs. This gene is expressed in a subtype of GABA‐vasoactive intestinal peptide (VIP) interneurons derived from the medial ganglionic eminence, suggesting that more differentiated cells are lost. *KCNK10* (TREK2) expression was significantly decreased, but was still significantly higher than in fetal NSCs.

We validated the expression of our candidate genes at the protein level using flow cytometry, before and after MACS enrichment (Figure 5F). The selected markers were expressed at the protein level by the majority of the cells (>90.7 ± 9%) and the expression was in general maintained or increased after CD133 purification with rare exceptions. MHCII was more variable, although in this case we cannot discard internalization. In contrast, TREK2 expression was maintained in most cells (97.5%‐95.8%) despite the decrease at the RNA level.

### 3.4 Transplantation study

To evaluate the safety of Gz‐NSCs, we first verified their trophic factor dependence (Figure S6A). Cells were expanded until passage 12—which corresponds to 22.25 ± 4.54 accumulated population doublings—and maintenance of normal karyotype was verified for the cell lines at this stage (Figure S6B). Recovery of purified and unpurified cells upon thawing and other cell growth characteristics relevant to scale‐up manufacturing were also analyzed and are shown in Figure S6.

**FIGURE 6:**
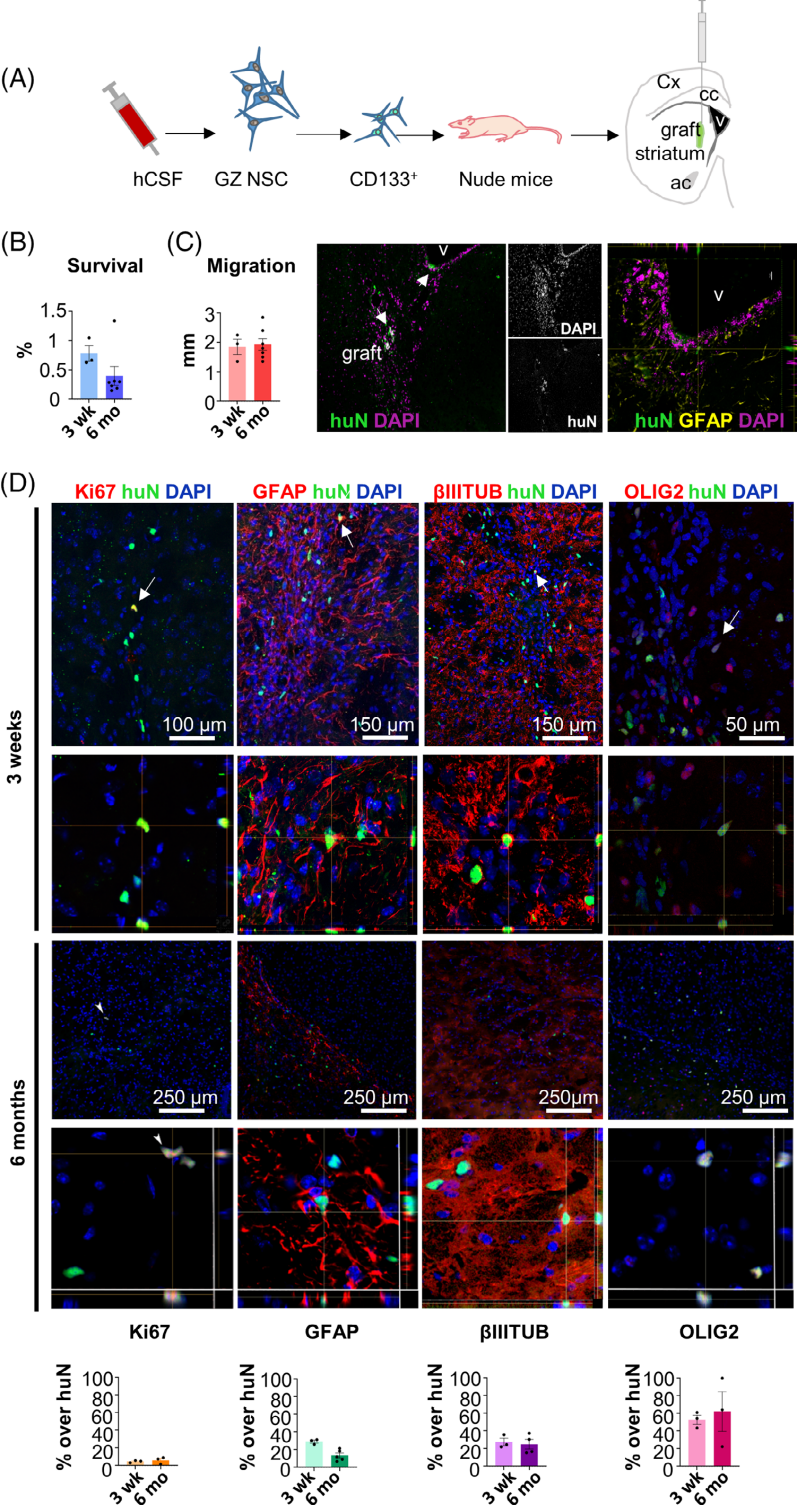
CD133^+^ purified Gz‐NSCs engraft into nude mice. A, Schematic representation of CD133^+^ purified Gz‐NSCs transplantation into the striatum of nude mice. B, Quantification of huN positive cells represented as the percentage over total transplanted cells. C, Migration of transplanted cells and a representative image showing engraftment of Gz‐NSCs at the striatum and subventricular zone (arrows). An orthogonal reconstruction shows a HuN positive cell in the SVZ. D, Immunofluorescence analysis of the expression of HuN (green) to identify human grafted cells and Ki‐67, β‐III‐tubulin, GFAP, and OLIG2 (in red). DAPI was used to stain nuclei. Shown are representative confocal sections, z‐stack orthogonal reconstructions demonstrating colocalization and quantification of the percentage of positive cells for each marker. V, ventricle

Next, we transplantedCD133^+^ purified cells in the striatum of nude mice (Figure 6A). None of the transplanted animals presented weight loss, neurological focal signs, or any adverse reactions for the duration of the study. Using a human‐specific antibody (huN), Gz‐NSCs were identified in the striatum of all animals at 3 weeks (n = 3) and 6 months (n = 7) after transplantation, albeit at low numbers (Figure 6B). Transplanted cells formed small grafts that did not cause anatomical distortion and a few migrated over the striatum and adjacent white matter tracts, occasionally reaching the VZ (Figure 6C). Transplanted cells showed low mitotic activity measured by Ki67 expression (4.59 ± 0.76 and 5.97 ± 2.22; Figure 6D). Phenotypic analyses showed the presence of human cells coexpressing GFAP, βIIItubulin or OLIG2 with huN (Figure 6D). At both time points, the cells appeared to predominantly adopt an oligodendrocyte fate.

## 4 DISCUSSION

We report here the isolation of a distinct class of NSCs, the Gz‐NSCs, from hemorrhagic CSF samples of premature neonates diagnosed with IVH grade IV. It is well established that NSCs from a given anatomical location give rise to corresponding regional cellular subtypes and, thus, display transcriptional and phenotypical differences with NSCs from other locations.^48^, ^49^, ^50^ We describe here features of Gz‐NSCs related to their regional and developmental origin in the ventral ganglionic eminences of the forebrain, which set them apart from other available human fetal forebrain NSC lines. Importantly, human interneuron neurogenesis continues into the third trimester of gestation, largely at the medial ganglionic eminence, which is also a source of oligodendrocyte precursors. Previous studies have shown that late neurogenesis is suppressed in premature births and that IVH arrests proliferation in the Gz at the level of the ganglionic eminences,^9^, ^11^ which could result in decreased GABA interneuron production and decreased myelination due to loss of oligodendrocyte precursors. These perturbations may contribute to persistent impairments in neurocognitive function in these children.

In addition to conspicuous differences in the expression of regional transcription factors, we report the expression of novel Gz‐NSC markers that differentiate these cells from fetal (dorsal) forebrain NSCs and were maintained after sorting for CD133^+^ cells, such as PODXL, IL1RAP, HLA‐DR, DLK1, and FZD5. These markers could serve to isolate and identify human Gz‐NSCs. However, more cases are required to validate these given that they can be developmentally regulated and our samples show some heterogeneity. PODXL is an interesting glycoprotein involved in apical polarity, which belongs to the CD34 family of syalomucins, whose absence has been reported to cause ventricular enlargement in mice.^51^ PODXL was expressed by nearly all cells in all samples and expression was maintained after CD133 sorting in all but one. The interleukin 1 receptor accessory protein (IL1RAP) is differentially expressed in the human VZ.^43^ We confirmed IL1RAP expression in Gz‐NSCs at the protein level by flow cytometry before and after MACS purification. Gz‐NSCs showed a variable expression of MHC class II proteins, which are developmentally expressed in proliferative brain areas (VZ and SVZ) at the level of the basal ganglia at mid‐gestational age (www.brainspan.org).^36^ Using IH, the existence of a population of MHC II/SOX2‐positive cells in the Gz of the human embryo, under no proinflammatory conditions, has been recently reported.^52^ According to that study, MHC II positive cells were present in different proportions at all developmental stages examined, coexpressed SOX2 and, like ours, did not express any microglial markers (data not shown). The biological relevance of MHC II expression in nonprofessional antigen presenting cells is not well understood but it has been suggested that may help modulate local immune responses and contribute to immunotolerance (reviewed in Reference 53). Nevertheless, we cannot rule out that, in addition to intrinsic developmental regulation, which has been demonstrated, albeit at slightly earlier stages (www.brainspan.org),^36^ increased levels of pro‐inflammatory cytokines like IFN‐γ and IL‐4 during the hemorrhage stage may induce and upregulate the transactivator CIITA and the MHC II pathway.^54^ Further studies are needed to explore this possibility, as it could have implications for future applications. Indeed, we cannot rule out that this contributed to the low *in vivo* survival in nude mice in our experiment compared with published rates of 40% for iPS‐NSCs or 20% for fetal NSCs.^38^

Gz‐NSCs showed a strong signal at the cell surface for FZD5, the putative receptor for Wnt5A, which is involved in neural specification and highly expressed in the VZ^55^ that was maintained in CD133^+^ Gz‐NSCs. Expression of the noncanonical Notch ligand DLK1, was also maintained in the sorted population. DLK1 is developmentally expressed in NSCs in the GEs and maybe involved in the regulation of the stem cell pool.^56^ On the other hand, TREK2 (*KCNK10*) a potassium channel that has been reported to be expressed by the ependymal cells^57^ and upregulated together with GFAP under ischemic conditions in astrocytes^58^ was transcriptionally downregulated in sorted Gz‐NSCs, yet the protein was maintained. Collectively these data provide a distinctive Gz‐NSC signature that is not dependent on the contribution of more differentiated cell types or contaminating lineages in the starting samples.

There was a large variability in both CD24 and CD34 positive populations between samples but it will be necessary to increase the sample size in order to examine a possible correlation with developmental stage (age), weight, severity or any other individual parameter. While CD24 expression has been described in NSCs, the presence of CD34^+^ cells was intriguing. We included HSC in the transcriptomic analysis to rule out a hematological origin of the CD133 population. In our data set, *CD31* (PECAM1), *CD53*, *CD37*, as well as typical leukocyte markers, such as selectin‐L or myeloperoxidase, and erythrocyte markers like fetal hemoglobin (data not shown), were not expressed by Gz‐NSCs, suggesting that hematopoietic and endothelial stem cells were not present in this population. Indeed, although it is usually considered a marker of hematopoietic and endothelial progenitor cells,^59^, ^60^ CD34 is also expressed in other lineages, such as the ependymal cells.^61^ Because ependymal cells are born in this region at a later developmental stage and ependymal loss causes a dysregulation of CSF homeostasis contributing to posthemorrhagic hydrocephalus, it will be interesting to study whether the expression of CD34 correlates with the capacity to generate this phenotype in vivo. In fact, after sorting we observed that CD34^+^ cells tended to remain in the negative fraction, suggesting that they may represent a more mature phenotype, perhaps in the ependymal lineage.

The shedding of neural progenitors and/or NSCs into the CSF of IVH and PHH patients had been previously suggested.^10^, ^62^ Our data partly support those studies, although, unlike Kruegger et al, we were not able to isolate NSCs from nonhemorrhagic CSF samples and it is likely that cells obtained by these authors from indwelling ventricular catheters represent a different population. Currently, stem cell therapies based on allogeneic umbilical cord‐derived cells are being tested in preclinical and clinical trials.^63^, ^64^, ^65^, ^66^, ^67^ These cells help to attenuate the effects associated to inflammation but, so far, no capability to replace lost tissue has been demonstrated. Although more studies are required, neuroendoscopic removal of the ventricular CSF in the hemorrhagic stage could be indicated in the future not only to ameliorate the problems associated with the accumulation of blood products and increased pressure, but also to isolate and bank Gz‐NSCs for the production of an autologous personalized cell‐therapy product directed to restore late neurogenesis and diminish long‐term neurological deficits. In this regard, we performed preliminary safety studies in which transplantation of Gz‐NSC purified CD133^+^ cells into the striatum of nude mice resulted in small neural grafts with no tumor formation or adverse reaction. Moreover, although survival in nude mice was very limited, a significant proportion of the grafted cells at 6 months expressed OLIG2, suggesting that Gz‐NSC treatment in an autologous setting could be a safe approach to restore oligodendrogenesis. Collectively our data demonstrate that Gz‐NSCs are a distinct population, which can be expanded, purified, and cryopreserved maintaining their ventral identity and a favorable safety profile. Therefore, we believe that Gz‐NSCs could be an optimal source to develop a cell therapy for preterm infants with IVH, for whom other cell therapies are already being tested.^68^

## 5 CONCLUSION

Here we describe a novel class of NSCs, the Gz‐NSCs that can be easily and robustly isolated from the CSF of preterm neonates with grade IV IVH undergoing neuroendoscopic lavage. We found that these cells, while being similar to fetal forebrain NSCs, have several distinctive hallmarks related to their regional and developmental origin in the ventral forebrain. Gz‐NSCs can be expanded and cryopreserved showing in vitro and in vivo differentiation potential, and pose no ethical concerns as the fluid is usually discarded. Thus, Gz‐NSCs could represent an optimal source for the development of an autologous cell therapy for infants with IVH, as well as a useful tool for studying the late stages of human neural development.

## CONFLICT OF INTEREST

B.F.M., E.G.M., R.S.P., and J.M. are authors of a patent application for the use of CSF‐NSCs (nº application European Patent Office: 200930943).

The other authors indicated no potential conflicts of interest.

## AUTHOR CONTRIBUTIONS

B.F.‐M.: conception and design, collection and assembly of data, data analysis and interpretation, manuscript writing; C.R.‐V.: collection and assembly of data, data analysis and interpretation; D.F.: collection of data, data analysis and interpretation; J.A.‐A., M.Á.M., R.C.‐C., M.M.‐L., D.C.P., M.F.B., A.G.: collection of data; L.L.‐N.: assembly and analysis of data, administrative support; E.G.‐M.: conception and design, collection of data; J.M.‐R.: conception and design, provision of study material or patients, collection of data, data analysis and interpretation; R.S.‐P.: conception and design, financial support, assembly of data, data analysis and interpretation; All authors: revision and final approval of manuscript.

## Supporting information


**Figure S1.** Supporting information

**Figure S2.** Supporting information

**Figure S3.** Supporting information

**Figure S4.** Supporting information

**Figure S5.** Supporting information

**Figure S6.** Supporting information

**Figure S7.** Supporting information

**Video S1.** Supporting information

**Table S1.** Antibodies

**Table S2.** Primers used for RT‐PCR.

**Table S3.** Flow cytometry results.

## Data Availability

The data that support the findings of this study are available upon reasonable request to the corresponding author.
