# Supplementary figures and images for: Retrieval of germinal zone neural stem cells from the cerebrospinal fluid of premature infants with intraventricular hemorrhage
Source: Stem Cells Transl Med. 2020 May 30;9(9):1085–101. doi: 10.1002/sctm.19-0323 (PMC7445027; doi:10.1002/sctm.19-0323)

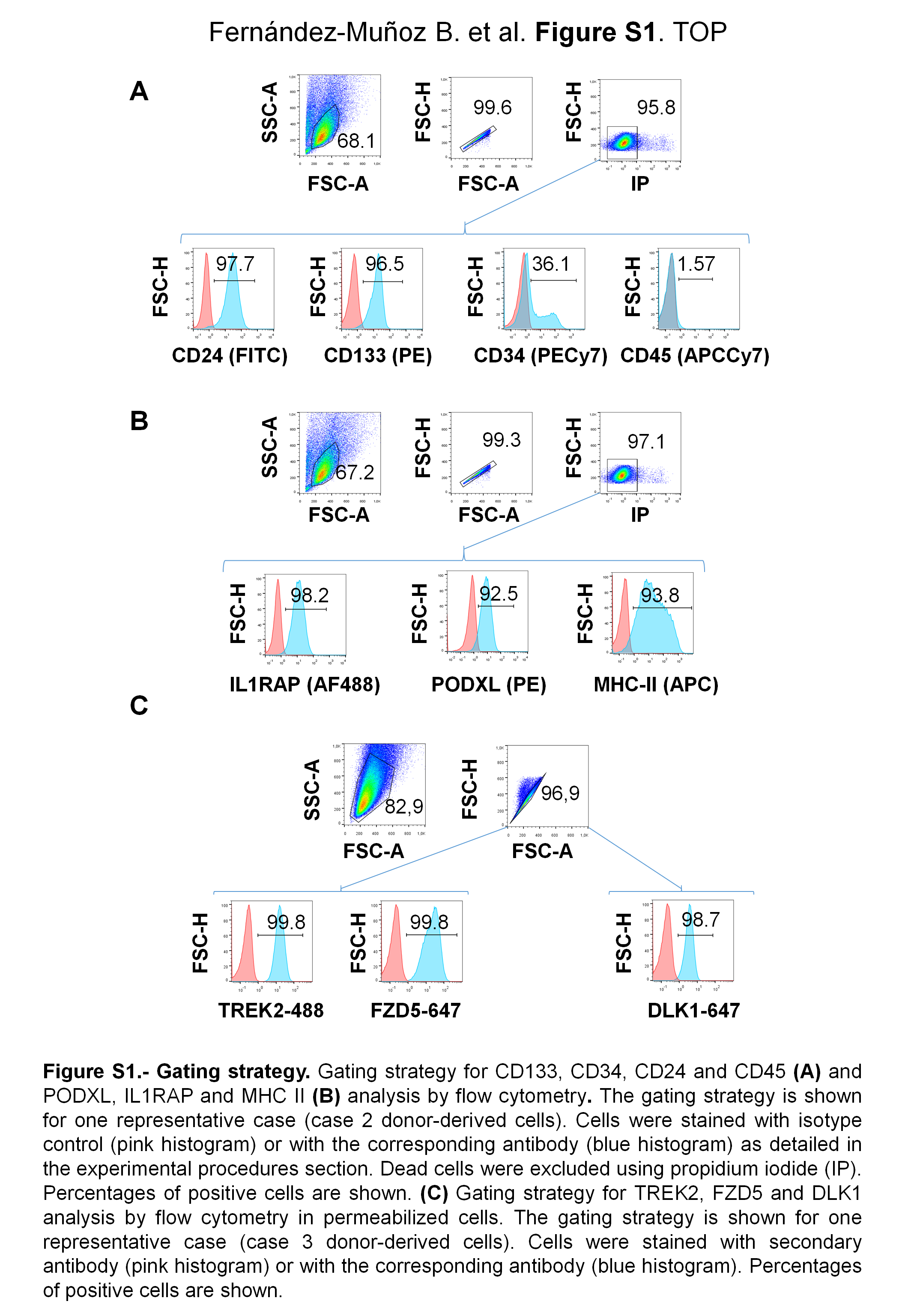

Supplement: Supplementary file 1 — Figure S1. Supporting information [file SCT3-9-1085-s003.tif]

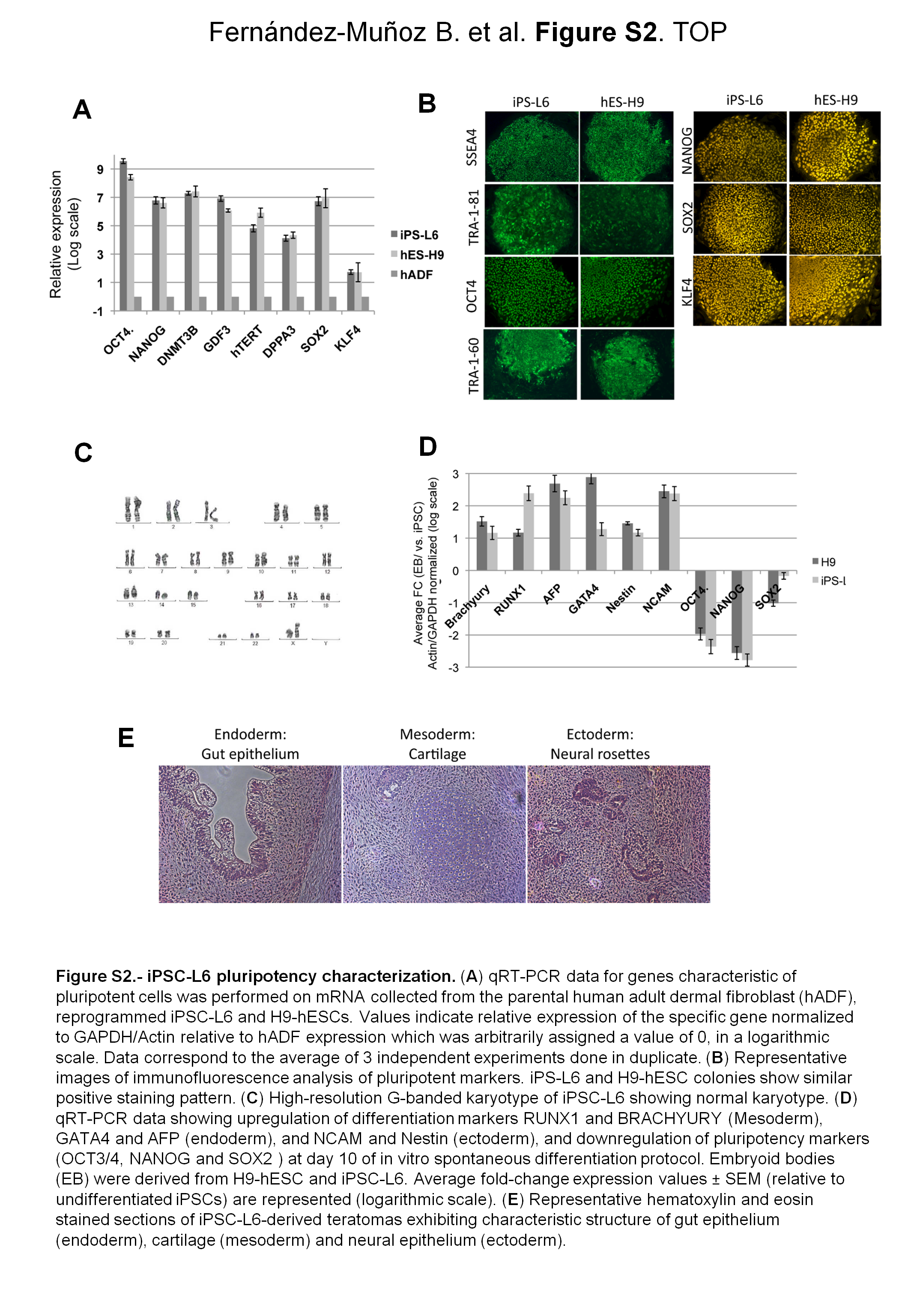

Supplement: Supplementary file 2 — Figure S2. Supporting information [file SCT3-9-1085-s004.tif]

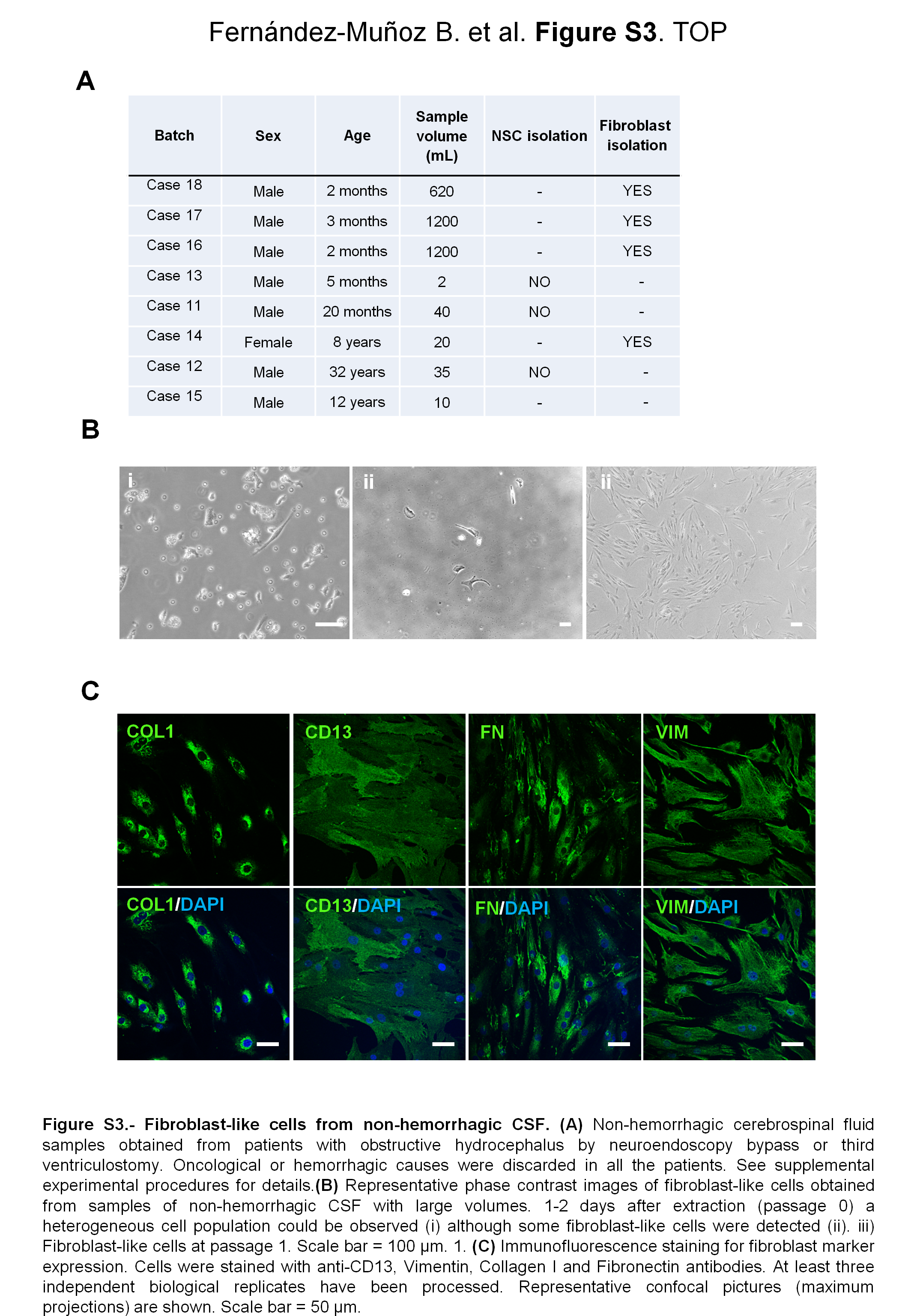

Supplement: Supplementary file 3 — Figure S3. Supporting information [file SCT3-9-1085-s005.tif]

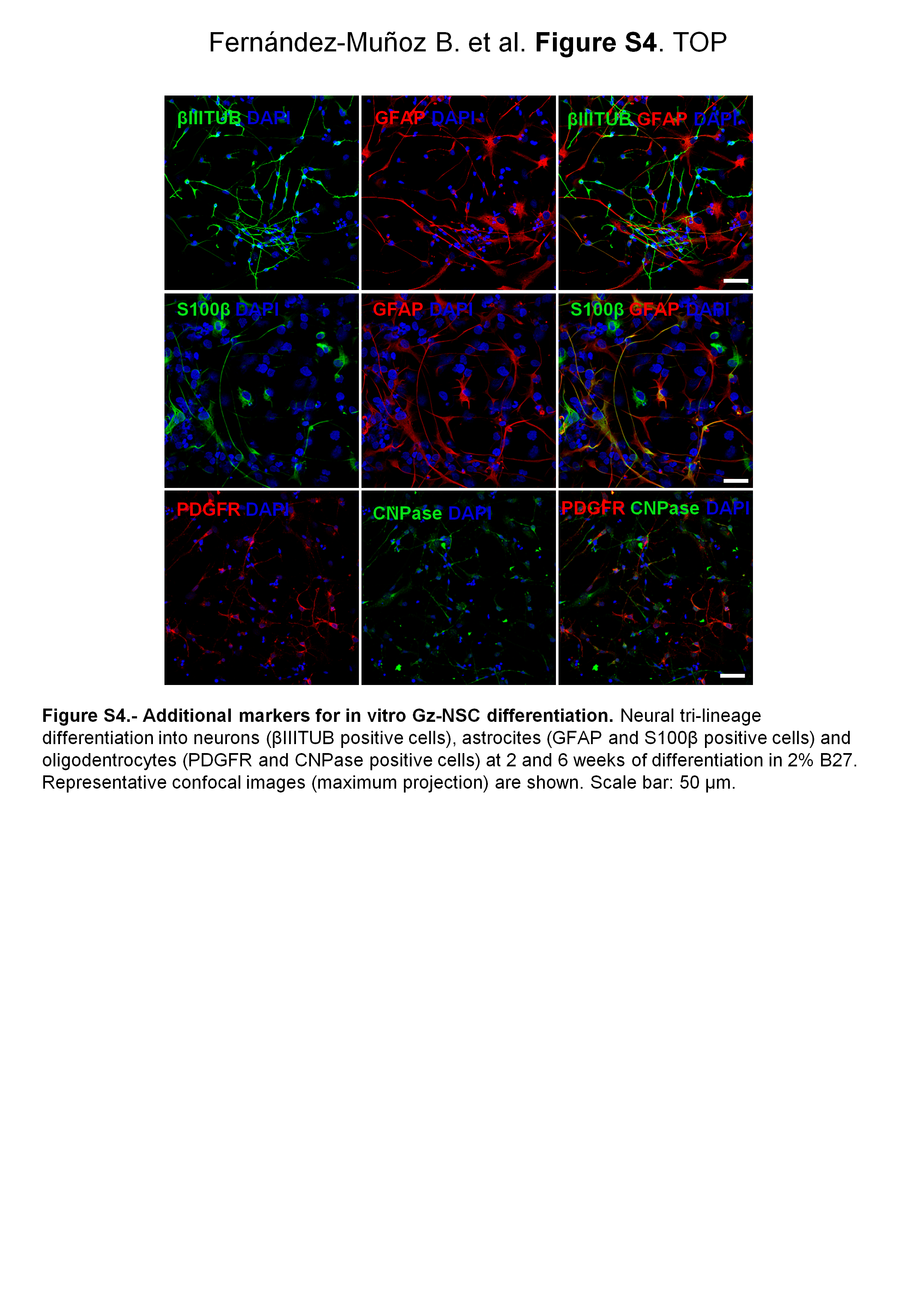

Supplement: Supplementary file 4 — Figure S4. Supporting information [file SCT3-9-1085-s006.tif]

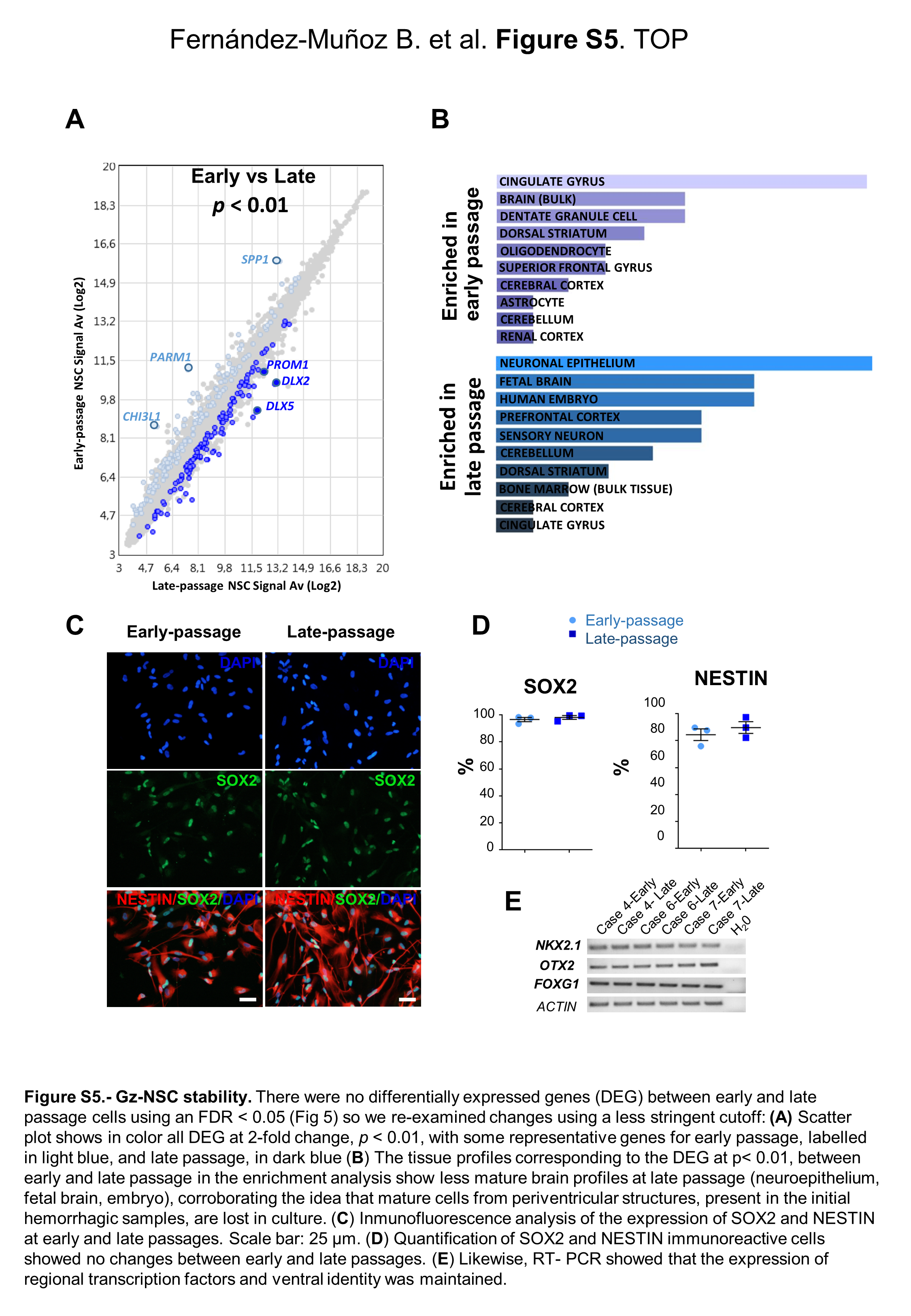

Supplement: Supplementary file 5 — Figure S5. Supporting information [file SCT3-9-1085-s007.tif]

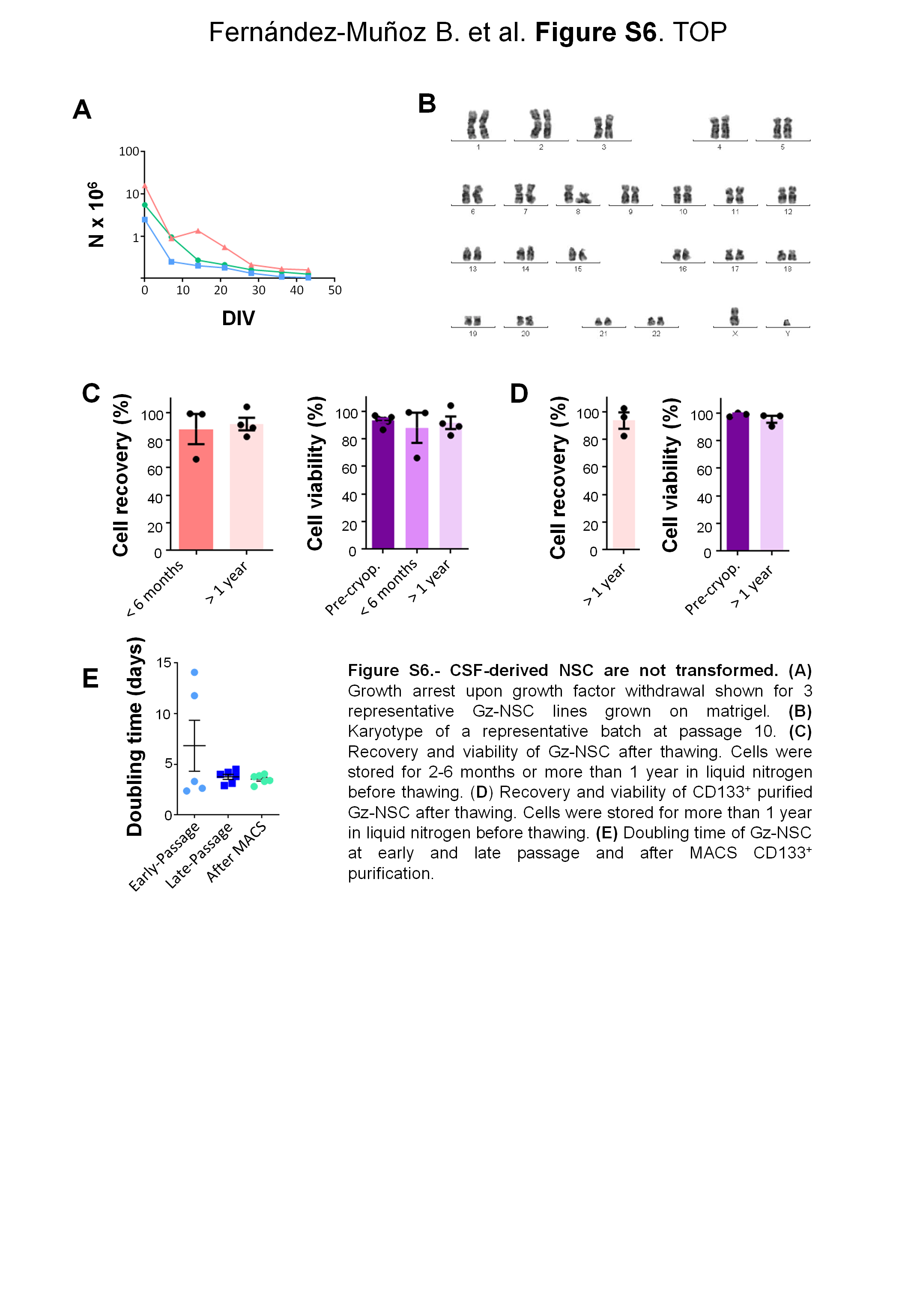

Supplement: Supplementary file 6 — Figure S6. Supporting information [file SCT3-9-1085-s008.tif]

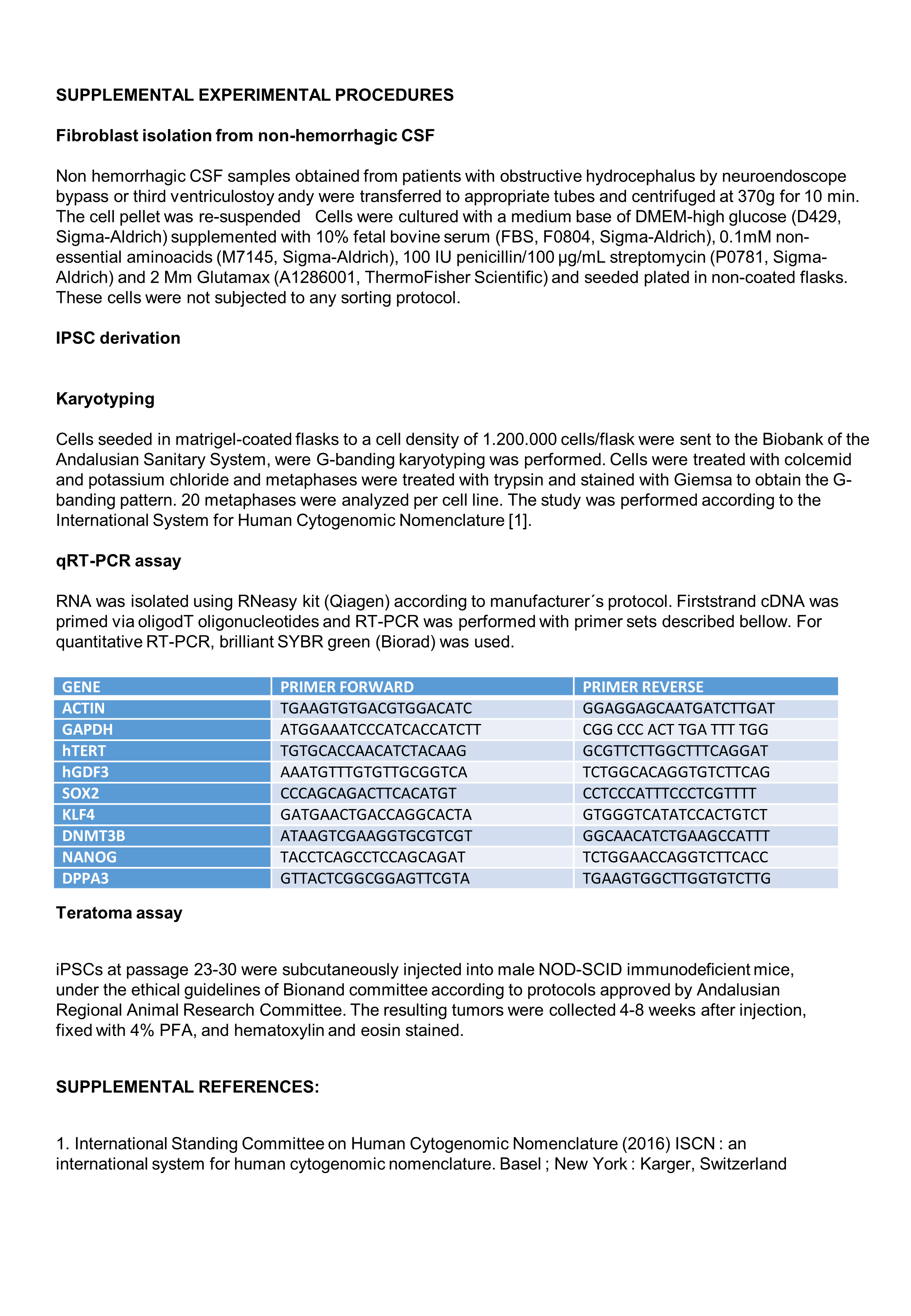

Supplement: Supplementary file 7 — Figure S7. Supporting information [file SCT3-9-1085-s009.tif]
