# Supplementary material for: Retrieval of germinal zone neural stem cells from the cerebrospinal fluid of premature infants with intraventricular hemorrhage
Source: Stem Cells Transl Med. 2020 May 30;9(9):1085–101. doi: 10.1002/sctm.19-0323 (PMC7445027; doi:10.1002/sctm.19-0323)
Supplement: Supplementary file 9 — Table S1. Antibodies [file SCT3-9-1085-s011.docx]

Fernández-Muñoz B. et al. **Table S1**. TOP

| **Primary antibodies** | **Dilution** | **Company (Catalog number)** |
| --- | --- | --- |
| SOX2 | 1:500 | Chemicon (ab5603) |
| NESTIN | 1:1000 | Abcam (ab22035) |
| NKX2.1 | 1:100 | R&D (MAB94581) |
| OTX2 | 1:100 | Neuromics (GT15095) |
| β-III TUBULIN | 1:1000 | Biolegend (801202) |
| GFAP (rabbit) | 1:2000 | Millipore (ab5804) |
| GFAP (chicken) | 1:1000 | Abcam (ab4674) |
| OLIG2 | 1:500 | Millipore (MABN50) |
| DCX | 1:500 | Abcam (ab18723) |
| CD13 | 1:200 | BD Bioscience (555393) |
| Collagen I | 1:500 | Abcam (ab34710) |
| Fibronectin | 1:500 | ThermoFisher (MS-165-P0) |
| Ki67 | 1:100 | Dako (M7240) |
| Vimentin | 1:1000 | Abcam (AB20346) |
| Frizzled-5 | 1:200 | Novus (NBP2-37451) |
| TREK2 | 1:100 | Alomone labs (APC-055) |
| DLK1 | 1:200 | ThermoFisher (MA5-15915) |
| PPLP4 | 1:10 | ThermoFisher (PA5-60944) |
| HuNu | 1:300 | Millipore |
| S100β | 1:100 | Abcam (ab52642) |
| PDGFRα | 1:100 | R&D (AF-307-NA) |
| IL-1 RAcP | 1:200 | Novus (NB1-76464SS) |
| CIPAR-1 (*PARM1*) | 1:500 | Novus (NBP1-85610) |
| **Antibodies conjugated to fluorochromes** | **Dilution** | **Company (Cat)** |
| CD133-PE | 1:11 | Miltenyi (130-098-046) |
| CD24-FITC | 1:11 | Miltenyi (130-099-118) |
| CD34-PECy7 | 1:20 | Biolegend (343516) |
| CD45-APCCy7 | 1:20 | Biolegend (368516) |
| Podocalixyn-PE | 1:11 | R&D (FAB1658P) |
| IL1RAP-AF488 | 1: 11 | R&D (FAB676G) |
| MHC-II-APC | 1:11 | Miltenyi (130-104-870) |
| **Secondary antibodies** | **Dilution** | **Company (Cat)** |
| Donkey Anti-Rabbit IgG (H+L) (Alexa -Fluor 488, green) | 1:200 | Invitrogen (A21206) |
| Donkey Anti-Rabbit IgG (H+L) (Alexa -Fluor 594, red) | 1:200 | Invitrogen (A11012) |
| Donkey Anti-Goat IgG (H+L) (Alexa-Fluor 594, green) | 1:200 | Invitrogen (A11058) |
| Donkey Anti-Mouse IgG (H+L) (Alexa-Fluor 594, red) | 1.200 | Invitrogen (A11005) |
| Donkey Anti-Mouse IgG (H+L) (Alexa-Fluor 488, green) | 1:200 | Invitrogen (A21206) |
| Goat Anti-Chicken IgY (H+L (Alexa-Fluor 594, red) | 1:200 | Invitrogen (A32759) |

**Table S1.- Antibodies.**
