# Supplementary material for: Retrieval of germinal zone neural stem cells from the cerebrospinal fluid of premature infants with intraventricular hemorrhage
Source: Stem Cells Transl Med. 2020 May 30;9(9):1085–101. doi: 10.1002/sctm.19-0323 (PMC7445027; doi:10.1002/sctm.19-0323)
Supplement: Supplementary file 10 — Table S2. Primers used for RT‐PCR. [file SCT3-9-1085-s001.docx]

Fernández-Muñoz B. et al. **Table S2**. TOP

| **Gene** | **Primer Sequence** | **Reference** |
| --- | --- | --- |
| CD133 | F: 5’-CACCGCTCTAGATACTGCTGTTGA-3’  R: 5’-TGATGGACCATGGACTATAACGTG-3’ | This work |
| SOX2 | F: 5’-AGAAGAGGAGAGAGAAAGAAAGGGAGAGA-3’  R:5’-GAGAGAGGCAAACTGGAATCAGGATCAAA-3’ | This work |
| FABP7 | F: 5’-AAGGATGGTGGAGGCTTTCT-3’  R: 5’-TTTGGTCACATTTCCCACCT-3’ | [1] |
| DCX | F: 5’-CATCCCCAACACCTCAGAAG-3’  R: 5’-GGAGGTTCCGTTTGCTGA-3’ | This work |
| MAP2 | F: 5’-CTAACCGAGGAAGCATTG-3’  R: 5’-TTCTCCTGCAACTATTCAAG-3’ | This work |
| SOX1 | F: 5’-ATTATTTTGCCCGTTTTCCC-3’  R: 5’-TCAAGGAAACACAATCGCTG-3’ | [1] |
| GFAP | F: 5´-TCTCTCGGAGTATCTGGGAACTG-3´  R: 5'- TTCCCTTTCCTGTCTGAGTCTCA-3´ | [1] |
| FOXG1 | F: 5'-TTCAGCTACAACGCGCTCAT-3'  R: 5'-ACAGATTGTGGCGGATGGAG-3' | [1] |
| OTX2 | F: 5´-TACCAATGCAGTCACCAGCCATCT-3´  R: 5'- TTCCAGGAGGCAGTTTGGTCCTTA-3´ | [1] |
| PAX6 | F: 5´-CCGGCAGAAGATTGTAGAGC-3´  F: 5´-CGTTGGACACGTTTTGATTG-3´ | [1] |
| NKX2.1 | F: 5´- AGCACACGACTCCGTTCTCA -3´  F: 5´- CCCTCCATGCCCACTTTCTT-3´ | This work |
| VAX1 | F: 5′- CTCTCCGAGACCCAGGTG-3′  R: 5′- GACACCACCGAGCGTAGC-3′ | [16] |
| SIX3 | F: 5′- CCGTCTCCGTTCCGAATTTG-3′  R: 5′- ACACATTTGCTATTTCCCTCTCC-3′ | [18] |
| SPP1 | F: 5′- TTGCAGCCTTCTCAGCCAA-3′  R: 5′- GGAGGCAAAAGCAAATCACTG-3′ | [2] |
| GSX2 | F: 5′- TGTCTCGACTCCGGAGGATT-3′  F: 5′- TTCACTCGGCGGTTCTGAAA-3′ | [10] |
| PARM1 | F: 5′-GCTCATAGACATGGAGACCACC-3′  R: 5′-CCCAGGACCCGTAGTCATGG-3′ | [6] |
| DLK-1 | F: 5’-AAGGACTGCCAGAAAAAGGAC-3’  R: 5’-GCAGAAATTGCCTGAGAAGC-3’ | [14] |
| CIITA | F: 5′-CCTGCTGTTCGGGACCTAAA-3′  R: 5′-GGATCCGCACCAGTTTGG-3′ | [9] |
| GLAST | F: 5′-TCCGTAAACGCACACTTTTG-3′  R: 5′-AAGTTCCCCAGGAAAGGAGA-3′ | This work |
| PODXL | F: 5’-CTCACCGGGGACTACAACC-3’  R: 5’-GCCTCCTCTAGCCACGGTA-3′ | [15] |
| PLPP4 | F: 5’-TTTGGATCCGTTCCAGAGAG-3’  R: 5’- CAGGGGTGTGAGGAAAGAAA-3’ | [17] |
| KCNK10 | F: 5'-AAGCATGGGCAGGGTGCGTC-3'  R: 5'-TCCGGCTCCCGGTCTTTGGT-3' | [11] |
| FZD5 | F: 5´-TGTCTGCTCTTCTCGGC-3´  R: 5´-CCGTCCAAAGATAAACTGCT-3´ | [3] |
| IL1RAP | F: 5'-GGGACTAGACACCATGAGGCAAAT-3'  R: 5'-TGCCTAGTCCAATACCAGATCAGAG-3' | [12] |
| HLA-DRA | F: 5’-GCTATCAAAGAAGAACATGTG-3’  R: 5’- GAGCGCTTTGTCATATTTCCAG-3’ | [13] |
| HLA-DQA1 | F: 5’-GAGCAGTTCTACGTGGACCTGG-3’  R: 5’-GGAACCTCATTGGTAGCAGCA-3’ | [5] |
| HLA-DPA1 | F: 5’-TGGCTGACTGAATTGCTGAC-3’;  R:5’ TGAGGGGTTCTTCAAAGGAG-3’ | [4] |
| ACTIN | F: 5’-TGAAGTGTGACGTGGACATC-3’  R: 5’-GGAGGAGCAATGATCTTGAT-3’ | [7] |

## **Table S2.- Primers used for RT-PCR.**

# SUPPLEMENTAL REFERENCES

1. Aguila JC, Blak A, van Arensbergen J, Sousa A, Vazquez N, Aduriz A, Gayosso M, Lopez Mato MP, Lopez de Maturana R, Hedlund E, Sonntag K-C, Sanchez-Pernaute R (2014) Selection Based on FOXA2 Expression Is Not Sufficient to Enrich for Dopamine Neurons From Human Pluripotent Stem Cells. Stem Cells Transl Med 3:1032–1042. doi: 10.5966/sctm.2014-0011

2. Bahrambeigi V, Salehi R, Hashemibeni B, Esfandiari E (2012) Transcriptomic comparison of osteopontin, osteocalcin and core binding factor 1 genes between human adipose derived differentiated osteoblasts and native osteoblasts. Adv Biomed Res 1:8. doi: 10.4103/2277-9175.94431

3. Blumenthal A, Ehlers S, Lauber J, Buer J, Lange C, Goldmann T, Heine H, Brandt E, Reiling N (2006) The Wingless homolog WNT5A and its receptor Frizzled-5 regulate inflammatory responses of human mononuclear cells induced by microbial stimulation. Blood 108:965–973. doi: 10.1182/blood-2005-12-5046

4. Conte M, Dell’Aversana C, Benedetti R, Petraglia F, Carissimo A, Petrizzi VB, D’Arco AM, Abbondanza C, Nebbioso A, Altucci L (2015) HDAC2 deregulation in tumorigenesis is causally connected to repression of immune modulation and defense escape. Oncotarget 6:886–901. doi: 10.18632/oncotarget.2816

5. Fernandez S, Wassmuth R, Knerr I, Frank C, Haas JP (2003) Relative quantification of HLA-DRA1 and -DQA1 expression by real-time reverse transcriptase-polymerase chain reaction (RT-PCR). Eur J Immunogenet Off J Br Soc Histocompat Immunogenet 30:141–148

6. Fladeby C, Gupta SN, Barois N, Lorenzo PI, Simpson JC, Saatcioglu F, Bakke O (2008) Human PARM-1 is a novel mucin-like, androgen-regulated gene exhibiting proliferative effects in prostate cancer cells. Int J cancer 122:1229–1235. doi: 10.1002/ijc.23185

7. Gonzalez-Munoz E, Arboleda-Estudillo Y, Otu HH, Cibelli JB (2014) Cell reprogramming. Histone chaperone ASF1A is required for maintenance of pluripotency and cellular reprogramming. Science 345:822–825. doi: 10.1126/science.1254745

9. Landmann S, Muhlethaler-Mottet A, Bernasconi L, Suter T, Waldburger JM, Masternak K, Arrighi JF, Hauser C, Fontana A, Reith W (2001) Maturation of dendritic cells is accompanied by rapid transcriptional silencing of class II transactivator (CIITA) expression. J Exp Med 194:379–391. doi: 10.1084/jem.194.4.379

10. Nadadhur AG, Leferink PS, Holmes D, Hinz L, Cornelissen-Steijger P, Gasparotto L, Heine VM (2018) Patterning factors during neural progenitor induction determine regional identity and differentiation potential in vitro. Stem Cell Res 32:25–34. doi: 10.1016/j.scr.2018.08.017

11. Park K-S, Han MH, Jang HK, Kim K-A, Cha E-J, Kim W-J, Choi YH, Kim Y (2013) The TREK2 Channel Is Involved in the Proliferation of 253J Cell, a Human Bladder Carcinoma Cell. Korean J Physiol Pharmacol 17:511–516. doi: 10.4196/kjpp.2013.17.6.511

12. Proulx M, Safoine M, Mayrand D, Aubin K, Maux A, Fradette J (2016) Impact of TNF and IL-1β on capillary networks within engineered human adipose tissues. J Mater Chem B 4:3608–3619. doi: 10.1039/C6TB00265J

13. Saikh KU, Kissner T, Ulrich RG (2002) Regulation of HLA-DR and co-stimulatory molecule expression on natural killer T cells by granulocyte-macrophage colony-stimulating factor. Immunology 106:363–372

14. Sakajiri S, O’kelly J, Yin D, Miller CW, Hofmann WK, Oshimi K, Shih L-Y, Kim K-H, Sul HS, Jensen CH, Teisner B, Kawamata N, Koeffler HP (2005) Dlk1 in normal and abnormal hematopoiesis. Leukemia 19:1404–1410. doi: 10.1038/sj.leu.2403832

15. Snyder KA, Hughes MR, Hedberg B, Brandon J, Hernaez DC, Bergqvist P, Cruz F, Po K, Graves ML, Turvey ME, Nielsen JS, Wilkins JA, McColl SR, Babcook JS, Roskelley CD, McNagny KM (2015) Podocalyxin enhances breast tumor growth and metastasis and is a target for monoclonal antibody therapy. Breast Cancer Res 17:46. doi: 10.1186/s13058-015-0562-7

16. Wataya T, Ando S, Muguruma K, Ikeda H, Watanabe K, Eiraku M, Kawada M, Takahashi J, Hashimoto N, Sasai Y (2008) Minimization of exogenous signals in ES cell culture induces rostral hypothalamic differentiation. Proc Natl Acad Sci U S A 105:11796–11801. doi: 10.1073/pnas.0803078105

17. Zhang X, Zhang L, Lin B, Chai X, Li R, Liao Y, Deng X, Liu Q, Yang W, Cai Y, Zhou W, Lin Z, Huang W, Zhong M, Lei F, Wu J, Yu S, Li X, Li S, Li Y, Zeng J, Long W, Ren D, Huang Y (2017) Phospholipid Phosphatase 4 promotes proliferation and tumorigenesis, and activates Ca2+-permeable Cationic Channel in lung carcinoma cells. Mol Cancer 16:147. doi: 10.1186/s12943-017-0717-5

18. Zheng Y, Zeng Y, Qiu R, Liu R, Huang W, Hou Y, Wang S, Leng S, Feng D, Yang Y, Wang Y (2018) The Homeotic Protein SIX3 Suppresses Carcinogenesis and Metastasis through Recruiting the LSD1/NuRD(MTA3) Complex. Theranostics 8:972–989. doi: 10.7150/thno.22328
