# Supplementary material for: Retrieval of germinal zone neural stem cells from the cerebrospinal fluid of premature infants with intraventricular hemorrhage
Source: Stem Cells Transl Med. 2020 May 30;9(9):1085–101. doi: 10.1002/sctm.19-0323 (PMC7445027; doi:10.1002/sctm.19-0323)
Supplement: Supplementary file 11 — Table S3. Flow cytometry results. [file SCT3-9-1085-s002.docx]

Fernández-Muñoz B. et al. **Table S3**. TOP

**Table S3. Flow cytometry results.**

| **Batch^a^** | | **CD133^+^** | **CD24^+^** | **CD34^+^** | **CD133^+^CD24^+^** | **CD133^+^CD34^+^** |
| --- | --- | --- | --- | --- | --- | --- |
| Case 2 | P3 | 42,75 | 18,73 | 3,1 | 9,74 | 0,3 |
|  | P7 | 80,60 | 91,17 | 66,05 | 74,26 | 49,07 |
|  | Fold change | 1,89 | 4,87 | 21,31 | 7,62 | 163,57 |
| Case 3 | P3 | 77,47 | 16,7 | 3,06 | 14,81 | 1,81 |
|  | P7 | 54,51 | 47,77 | 8,66 | 36,51 | 5,47 |
|  | Fold change | 0,70 | 2,86 | 2,83 | 2,47 | 3,02 |
| Case 4^b^ | P3 | - | - | - | - | - |
|  | P7 | 48,69 | 96,3 | 62,95 | 48,02 | 27,83 |
|  | Fold change | - | - | - | - | - |
| Case 5^c^ | P3 | 34,94 | 30,3 | 16,07 | 5,73 | 1,17 |
|  | P7 | 3,25 | 5,12 | 34,18 | 1 | 0,36 |
|  | Fold change | 0,09 | 0,17 | 2,13 | 0,07 | 0,13 |
| Case 6 | P3 | 44,83 | 81,63 | 21,54 | 38,02 | 2,98 |
|  | P7 | 88,02 | 90,56 | 2,57 | 83,02 | 2,29 |
|  | Fold change | 1,96 | 1,11 | 0,12 | 2,18 | 0,77 |
| Case 7 | P3 | 88,02 | 57,88 | 27,45 | 49,57 | 20,11 |
|  | P7 | 77,57 | 60,43 | 37,17 | 43,4 | 22,4 |
|  | Fold change | 0,88 | 1,04 | 1,35 | 0,88 | 1,11 |
| Case 8 | P3 | 65,94 | 44,48 | 25,26 | 33,5 | 15,53 |
|  | P7 | 52,84 | 14,37 | 2,64 | 11,32 | 0,82 |
|  | Fold change | 0,80 | 0,32 | 0,10 | 0,34 | 0,05 |

^a^ Only 7 samples were included in this analysis. There were no available cells from the first case for flow cytometry studies. The presence of NSC was examined by immunofluorescence.

^b^ Data at passage 3 are lacking because few cells were initially obtained from this case, but we were successful at expanding and analyze them at late passage.

^c^ CD133 expression dropped drastically with passages and therefore cells isolated from this sample (42 weeks old) were excluded from further analysis.
